# Rehabilitation Transition Program to Improve Community Participation Among Stroke Survivors: A Randomized Clinical Trial

**DOI:** 10.1001/jamanetworkopen.2024.37758

**Published:** 2024-10-07

**Authors:** Rebecca M. Bollinger, Melissa J. Krauss, Emily K. Somerville, Brianna M. Holden, Gabrielle Blenden, Holly Hollingsworth, Audrey A. Keleman, Alexandre Carter, Timothy D. McBride, Abigail R. Barker, Yan Yan, Susan L. Stark

**Affiliations:** 1 Program in Occupational Therapy, Washington University in St Louis School of Medicine, St Louis, Missouri; 2 Division of Neurorehabilitation, Department of Neurology, Washington University in St Louis School of Medicine, St Louis, Missouri; 3 Center for Advancing Health Services, Economics, and Policy Research, Institute for Public Health at Washington University in St Louis, St Louis, Missouri; 4 Department of Surgery, Washington University in St Louis School of Medicine, St Louis, Missouri

## Abstract

**Importance:**

Interventions are needed to support the long-term needs of stroke survivors when they transition from inpatient rehabilitation to home, where they face new home and community environmental barriers.

**Objective:**

To compare the efficacy of a novel, enhanced rehabilitation transition program with attentional control to improve community participation and activity of daily living (ADL) performance and to reduce environmental barriers in the home and community after stroke.

**Design, Setting, and Participants:**

This phase 2b, parallel randomized clinical trial assessed patients 50 years or older who had experienced an acute ischemic stroke or intracerebral hemorrhage, were independent in ADLs before stroke, and planned to be discharged home. Patients were assessed at an inpatient rehabilitation facility in St Louis, Missouri, and their homes from January 9, 2018, to December 20, 2023.

**Intervention:**

Community Participation Transition after Stroke (COMPASS), including home modifications and strategy training.

**Main Outcomes and Measures:**

The primary outcome was community participation (Reintegration to Normal Living Index). Secondary outcomes were daily activity performance (Stroke Impact Scale ADL domain and the In-Home Occupational Performance Evaluation [I-HOPE] activity, performance, and satisfaction scores) and environmental barriers in the home (I-HOPE environmental barriers score).

**Results:**

A total of 185 participants (mean [SD] age, 66.3 [9.0] years; 105 [56.8%] male) were randomized (85 to the COMPASS group and 100 to the control group). The COMPASS and control participants experienced similar improvements in community participation by 12 months, with no significant group (mean difference, 0.3; 95% CI, −4.6 to 5.2; *P* = .91) or group × time interaction (between-group differences in changes over time, 1.3; 95% CI, −7.1 to 9.6; *P* = .76) effects. Improvements in I-HOPE performance and satisfaction scores were greater for COMPASS participants than control participants at 12 months (between-group differences in changes for performance: 0.39; 95% CI, 0.01-0.77; P = .046; satisfaction: 0.52; 95% CI, 0.08-0.96; *P* = .02). The COMPASS participants had greater improvements for I-HOPE environmental barriers than the control participants (P = .003 for interaction), with the largest differences at 6 months (between-group differences in changes: −15.3; 95% CI −24.4 to −6.2).

**Conclusions and Relevance:**

In this randomized clinical trial of stroke survivors, participants in both groups experienced improvements in community participation. COMPASS participants had greater improvements in self-rated performance and satisfaction with performing daily activities as well as a greater reduction in environmental barriers than control participants. COMPASS reduced environmental barriers and improved performance of daily activities for stroke survivors as they transitioned from inpatient rehabilitation to home.

**Trial Registration:**

ClinicalTrials.gov Identifier: NCT03485820

## Key Points

**Question:**

Is a rehabilitation transition program more efficacious than attentional control for improving community participation and activity of daily living performance and for reducing environmental barriers in the home and community after stroke?

**Findings:**

In this randomized clinical trial of 185 stroke survivors, both rehabilitation transition program participants and control participants experienced improvements in community participation. Rehabilitation transition program participants had greater improvements in self-rated performance and satisfaction with daily activities and greater reduction in environmental barriers than attentional control participants.

**Meaning:**

A rehabilitation transition program may bridge the gap from inpatient rehabilitation to home by reducing environmental barriers and fostering quicker engagement in activities of daily living to improve stroke survivors’ health outcomes.

## Teaser

This randomized clinical trial of stroke survivors compares the efficacy of a rehabilitation transition program with attentional control to improve community participation.

## Introduction

Stroke is a leading cause of serious, long-term disability and death for more than 795 000 adults in the US annually.^1,2^ Stroke survivors often report difficulty performing activities of daily living (ADLs; eg, bathing and dressing),^3^ decreased quality of life,^4,5^ and reduced community participation.^6,7,8,9^ Stroke survivors have a high risk of long-term nursing care^10^ and a high rate of injurious falls.^11^ Solutions are needed to improve long-term outcomes for stroke survivors.

Stroke is fundamentally a chronic condition that is currently managed as an acute event.^12^ Hospital readmission is high after stroke^13,14^ and frequently attributed to lack of transitional planning.^15^ Approximately 25% of stroke survivors are discharged to inpatient rehabilitation (IR),^16,17,18^ which focuses on resuming ADLs but does not typically include home visits. Most stroke survivors (87%) leave IR facilities without the skills to successfully address new barriers when returning to the community.^19^ Stroke survivors may be referred to home health or outpatient therapy after stroke, but many report “waiting” at home 6 to 12 months after discharge, when they have the greatest potential for recovery, before resuming valued activities.^20,21^ Interventions to reestablish daily routines and improve community reintegration as stroke survivors transfer from medical to community models of care are lacking.

At home, stroke survivors face environmental barriers that make it difficult to complete daily activities. Reducing environmental barriers in the home can improve daily activity performance^22,23,24,25,26,27,28,29,30,31,32,33^ and reduce falls^34,35,36,37,38,39,40^ but has not been proven among stroke survivors. Resolving these barriers and improving community independence could significantly improve stroke survivors’ long-term health outcomes.

To address the gap in care as stroke survivors transition from IR to home, we developed Community Participation Transition After Stroke (COMPASS), a novel, enhanced rehabilitation transition program delivered at home. COMPASS includes home modifications and strategy training to help patients prioritize ADLs, identify barriers to performance, and develop strategies to resolve barriers. The study’s objectives were to compare the efficacy of COMPASS to attentional control (AC; stroke education) to improve community participation and ADL performance and reduce environmental barriers in the home and community after stroke.

## Methods

### Study Design and Procedures

This study was a phase 2b, parallel randomized clinical trial to examine the efficacy of COMPASS compared with AC to prepare for a phase 3 trial. Study procedures were approved by the Washington University in St Louis Institutional Review Board. The trial protocol can be found in Supplement 1. Participants’ written informed consent was obtained. The Consolidated Standards of Reporting Trials (CONSORT) reporting guideline was followed.^41^ The study protocol was previously published.^42^

Stroke survivors were recruited while transitioning from acute care to IR or during IR in St Louis, Missouri, from January 9, 2018, through January 10, 2023 (paused recruitment from March 16, 2020, to January 9, 2021, due to the COVID-19 pandemic). Referrals from an acute care stroke patient registry at the Washington University Medical Campus were prescreened for eligibility (stroke diagnosis, age ≥50 years, and planned discharge to IR facility) using electronic health records. Patients receiving IR from an outside hospital were also referred. Those who passed prescreening were approached in the IR facility by research staff. Eligible participants were 50 years or older, had experienced an acute ischemic stroke or intracerebral hemorrhage, were independent in ADLs before stroke (≤2 on the premorbid Modified Rankin Scale),^43^ and planned to be discharged home (verified with patient’s case manager or therapy team). Exclusion criteria were life expectancy of less than 6 months, significant cognitive impairment (>10 on the Short Blessed Test),^44^ moderate to severe aphasia (National Institutes of Health Stroke Scale best language score ≥2),^45^ residence more than 60 miles from the research laboratory, and planned discharge to an institutional setting.

Participants received a baseline in-home visit before discharge from an IR facility. An occupational therapist (OT; n = 4; mean of 5 years of experience) used the In-Home Occupational Performance Evaluation (I-HOPE) to establish baseline activity patterns and identify environmental barriers.^46^ During the COVID-19 pandemic, when patients were not allowed to leave the IR facility before discharge, the OT observed the participant performing daily activities during IR and used clinical reasoning to determine barriers when visiting the home without them. At the end of this visit, the OT randomized participants by age and Functional Independence Measure (FIM)^47^ score to COMPASS or AC using a 1:1 allocation ratio (15 total strata: 3 FIM strata and 5 age strata). This ratio was adjusted with unequal allocation probability at the study midpoint to increase participants randomized to AC due to significantly greater attrition in AC. The randomization sequence was developed a priori by the study statistician (Y.Y.) using a computerized formal probability model and concealed using Research Electronic Data Capture (REDCap) until randomization. The IR staff were blinded to allocation to prevent any influences of the intervention on treatment and discharge plans.

### Intervention and Control Conditions

COMPASS is guided by the International Classification of Functioning, Disability, and Health^48^ and a competence-press model,^49^ which posits that removing environmental barriers matched with the survivor’s functional loss will improve daily activity performance and participation.^49^ COMPASS is tailored to each participant’s goals, patterns of functional loss, and unique home and community environments. There are 2 essential components of COMPASS: home modifications and strategy training. Home modifications include changes to the physical environment, education to use it safely, and changing social support to compensate for environmental barriers, including (1) structural changes, (2) adaptive equipment, (3) task or behavior modifications, and (4) personal assistance. During strategy training, participants learn to create goals, make plans for improving their performance in self-selected problematic activities,^50,51,52^ and use guided discovery to self-monitor and evaluate their performance during activities.^52^ Throughout the intervention, the OT uses motivational enhancement to increase adherence to the intervention.^53,54^

For those randomized to COMPASS, during the baseline in-home visit, the OT collaborated with the participant to identify and prioritize problematic activities in the home and community. The OT used task analysis to identify barriers in the home and presented multiple solutions to the participant. Once they chose a solution, the OT obtained the modification and worked with a contractor as needed to install it. When possible, essential home modifications (eg, tub transfer benches and ramps) were installed before IR discharge. After returning home, the participant received 4 visits during which the OT provided additional environmental modifications. The OT demonstrated how to use the modifications or equipment, and the participant practiced with the new modifications. The OT also used strategy training with the participant to identify a desired community outing, brainstorm barriers and solutions, plan and complete the community outing while problem solving as needed, and brainstorm future community activities. In 2020, the community activity was removed due to the COVID-19 pandemic. A booster session (1-2 visits if needed) was conducted 4 to 5 months after the initial intervention visits.

The AC participants received stroke education^55^ provided by an OT practitioner in person during 4 sessions. Topics included symptoms of stroke, risk factors and preventing stroke recurrence, nutrition, managing emotions, sleep, fatigue, pain, and social support. Daily activities, environmental barriers, strategy training, and home modifications were not included.^56^ Booster sessions were offered in person or virtually 4 to 5 months after the initial education sessions.

### Data Collection and Measures

Assessments were collected by blinded OT raters at 3 time points after baseline: immediately after the intervention or AC visits, 6 months after stroke, and 12 months after stroke. For some participants, 12-month assessments were delayed a few months due to the COVID-19 pandemic.

The primary outcome was community participation measured by the Reintegration to Normal Living Index (RNLI).^57,58,59^ The self-reported, 11-item RNLI is reliable and valid and assesses personal satisfaction in mobility, self-care, family roles, and social and leisure activities. Item responses (range of 1 [does not describe me at all] to 10 [describes my situation fully]) were summed and converted for a total score with a maximum of 100; higher scores indicated greater community participation.

Secondary outcomes included daily activity performance and home environmental barriers. The Stroke Impact Scale ADL domain (SIS-ADL) was used to measure daily activity performance (range, 0-100, with higher scores indicating less difficulty).^60^ I-HOPE was used to assess activity performance, satisfaction, and environmental barriers.^46^ Participants sorted 44 activities into ability categories, and a composite score was calculated (range, 0-1, with higher scores indicating greater independence in daily activities). Participants then prioritized 10 or fewer activities and rated their performance (range of 1 [unable to perform] to 5 [able to perform without any difficulty]) and performance satisfaction (range of 1 [not satisfied at all] to 5 [completely satisfied]). Mean performance and satisfaction scores were calculated. Finally, barriers to performing activities were rated on severity (range of 0 [independent with/without a device] to 5 [no activity]) and summed; higher scores indicated greater barrier severity.

Participant characteristics collected at baseline included age, sex, race and ethnicity, stroke type, marital status, living situation (living alone or with someone else), depression (Geriatric Depression Scale–Short Form),^61^ FIM,^47^ number of comorbidities (up to 16), and number of days in IR. Participant characteristics were collected via self-report to describe the sample. After the intervention or AC, participants reported whether they received home health, outpatient therapy, or day therapy monthly via telephone for 12 months.

### Sample Size

The trial was powered to detect differences in mean changes between groups in primary and secondary outcomes with 80% power using 2-sided, 2-sample, unequal-variance *t* tests and α = .05. We estimated that mean (SD) changes in COMPASS and AC would be 15.3 (22.6) and 1.3 (23.4) for RNLI, 15.7 (16.1) and 5.6 (9.1) for SIS-ADL, and 62.1 (26.1) and 46.2 (18.8) for I-HOPE barrier severity scores, respectively.^62^ Total sample size estimates were 130 (65 per group) for RNLI, 84 (42 per group) for SIS-ADL, and 100 (50 per group) for I-HOPE. We planned to enroll 180 participants and expected 30% attrition, which would provide a large enough sample size for all outcomes. More details are available in the published protocol.^42^

### Statistical Analysis

Analyses were conducted using an intention-to-treat paradigm. Baseline characteristics were compared between study completers and dropouts using independent-sample *t* tests for continuous variables and χ^2^ tests for categorical variables. Linear mixed models were used to compare changes in outcomes between groups. Mixed-effects models allow for correlation among repeated measures within individuals and use all available data from each participant with estimates that are unbiased by missing data, assuming data are missing at random.^63^ The models included group, time point, and group × time interaction as fixed effects. The significance of the group × time interaction indicates significant differences in change over time between groups. For our primary outcome, we used baseline and 12-month scores. For secondary outcomes, models were first run with the inclusion of baseline and 12-month scores and then extended to include scores after intervention and 6 months after stroke. We included covariates (race, marital status, living situation, depression, and length of stay in IR facility) that may have strong associations with our outcomes^64,65,66,67,68,69,70,71^ as well as calendar time at baseline (in months). As a post hoc sensitivity analysis, each model was run additionally with the inclusion of baseline values of other outcomes. To test for specific comparisons of interest (eg, changes over time within each group), we used estimate statements. Model residuals were evaluated for normal distributions. All tests were 2-tailed. *P* < .05 was considered to be statistically significant. SPSS, version 29.0 (SPSS Inc) and SAS, version 9.4 (SAS Institute Inc) were used for analyses.

## Results

### Participant Flow

**Figure 1.**
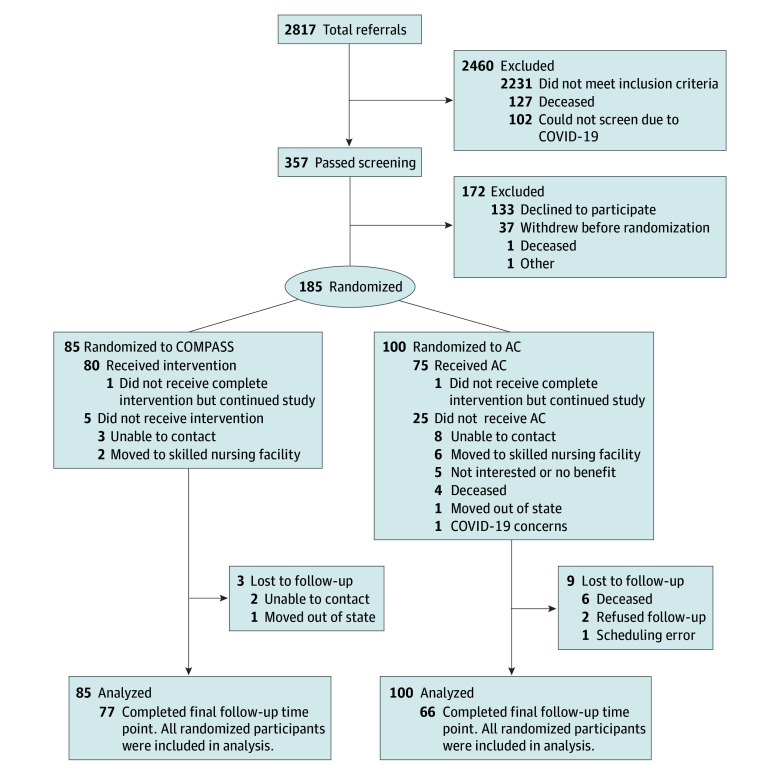
CONSORT Diagram AC indicates attentional control; COMPASS, Community Participation Transition After Stroke.

Of 2817 individuals with stroke in acute care, 357 were eligible for COMPASS (Figure 1), and 185 were randomized: 85 to COMPASS and 100 to AC. Among those randomized, 77 (90.6%) in the COMPASS group and 66 (66.0%) in the AC group completed the study. Rates of missing data at baseline and 12-month follow-up were 6.0% and 29.2% for RNLI and SIS-ADL, 7.6% and 25.4% for I-HOPE activity score, 11.4% and 26.5% for I-HOPE performance and satisfaction scores, and 11.4% and 27.6% for I-HOPE barrier severity scores, respectively. Baseline characteristics did not differ between dropouts (n = 42) and completers (n = 143) and those with missing vs complete outcome data. Within each treatment group, there were no significant differences in baseline values of outcomes between dropouts and completers. There was greater attrition after COVID-19 (28 of 85 [32.9%]) than before COVID-19 (14 of 100 [14.0%]; *P* = .002). Notably, 10 AC participants dropped out due to death. The mean (SD) time from baseline to 12-month follow-up was 12.3 (1.8) months.

### Baseline Characteristics

Characteristics for all randomized participants by treatment group are given in Table 1. The mean (SD) age was 66.3 (9.0) years; 105 (56.8%) were male and 80 (43.2%) female; and 108 (58.4%) were Black, 76 (41.1%) White, and 1 (0.5%) with unlisted race. A total of 148 (80.0%) experienced an ischemic stroke, and 58 (31.4%) lived alone. Similar proportions of COMPASS and AC participants received home health (37 of 78 [47.4%] in the COMPASS group and 35 of 74 [47.3%] in the AC group), outpatient therapy (46 of 78 [59.0%] in the COMPASS group and 40 of 72 [55.6%] in the AC group), and day therapy (17 of 75 [22.7%] in the COMPASS group and 13 of 71 [18.3%] in the AC group).

**Table 1.** Baseline Characteristics of All Randomized COMPASS and AC Participants

Characteristic	No. (%) of participants^a^	
	COMPASS group (n = 85)	AC group (n = 100)
Age, mean (SD), y	66.3 (9.0)	66.3 (9.0)
Sex		
Female	35 (41.2)	45 (45.0)
Male	50 (58.8)	55 (55.0)
Race		
Black	45 (52.9)	63 (63.0)
White	40 (47.1)	36 (36.0)
Unlisted	0	1 (1.0)
Marital status		
Married or partner	48 (56.5)	44 (44.0)
Single, divorced, or widowed	37 (43.5)	56 (56.0)
Living situation		
Lives alone	26 (30.6)	32 (32.0)
Lives with someone	59 (69.4)	68 (68.0)
Type of stroke		
Ischemic	70 (82.4)	78 (78.0)
Hemorrhagic	15 (17.6)	22 (22.0)
No. of comorbidities, mean (SD)^b^	3.5 (1.8)	3.5 (2.3)
Functional Independence Measure score, mean (SD)	76.1 (18.2)	79.5 (18.0)
Length of IR, mean (SD), d^c^	20.7 (16.5)	18.0 (7.8)
GDS-SF score, mean (SD)^d^	3.5 (2.7)	3.1 (2.8)
RNLI^e^	60.5 (21.8)	62.1 (20.9)
SIS-ADL score^e^	54.1 (21.3)	56.9 (22.8)
I-HOPE activity score^f^	0.72 (0.16)	0.74 (0.18)
I-HOPE performance score^g^	2.4 (0.7)	2.7 (0.8)
I-HOPE satisfaction score^g^	2.2 (0.8)	2.6 (1.0)
I-HOPE barrier severity score^g^	69.0 (31.5)	63.7 (36.5)

Abbreviations: AC, attentional control; COMPASS, Community Participation Transition After Stroke; GDS-SF, Geriatric Depression Scale–Short Form; I-HOPE, In-Home Occupational Performance Evaluation; IR, inpatient rehabilitation; RNLI, Reintegration to Normal Living Index; SIS-ADL, Stroke Impact Scale–Activities of Daily Living domain.

^a^ Unless otherwise indicated.

^b^ Sample sizes are 83 COMPASS and 95 AC participants for comorbidities data.

^c^ Sample sizes are 85 COMPASS and 98 AC participants for number of days in IR.

^d^ Sample sizes are 84 COMPASS and 96 AC participants for GDS-SF data.

^e^ Sample sizes are 83 COMPASS and 91 AC participants for RNLI and SIS-ADL data.

^f^ Sample sizes are 82 COMPASS and 89 AC participants for I-HOPE activity score data.

^g^ Sample sizes are 78 COMPASS and 86 AC participants for I-HOPE performance, satisfaction, and barrier data.

### Intervention Implementation

The 80 COMPASS participants who received the intervention had 1 predischarge home visit plus a mean (SD) of 5.5 (1.8) postdischarge visits. The 75 AC participants who received control visits had 1 predischarge home visit plus a mean (SD) of 4.3 (1.8) postdischarge visits. Seven hundred eighty home modification recommendations were made, the most common being adaptive equipment (536 [68.7%]) and architectural modifications (198 [25.4%]).

### Primary Outcome

**Figure 2.**
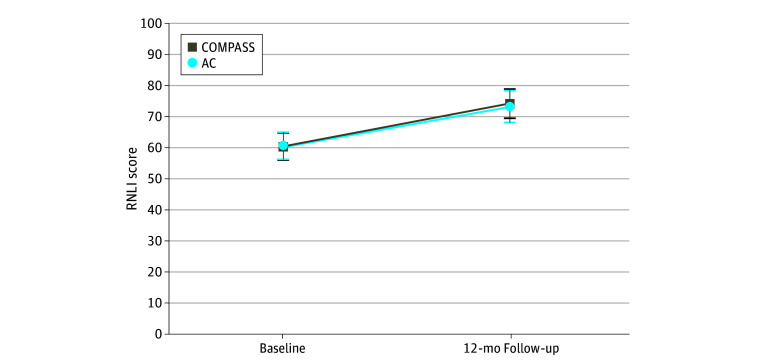
Estimated Marginal Means for Reintegration to Normal Living Index (RNLI) at Baseline and 12 Months Error bars indicate 95% CIs. AC indicates attentional control; COMPASS, Community Participation Transition After Stroke.

Table 2 gives the mixed-model results (using baseline and 12 months) adjusted for covariates. Figure 2 shows estimated marginal means. Significant improved community participation (RNLI) was observed between baseline and 12 months for both groups (mean improvement for COMPASS, 13.9; 95% CI, 8.1-19.8; mean improvement for AC, 12.7; 95% CI, 6.7-18.6). There were no significant group (mean difference, 0.3; 95% CI, −4.6 to 5.2; *P* = .91) or group × time interaction (between-group differences in changes over time, 1.3; 95% CI, −7.1 to 9.6; *P* = .76) effects.

**Table 2.** Results From Linear Mixed Models of Primary and Secondary Outcomes^a^

Outcome	Mean (SE)				Mean (95% CI)			
	COMPASS		AC		Between-group difference	Changes over time within each group		Between-group differences in changes over time
	Baseline	12-mo follow-up	Baseline	12-mo follow-up		COMPASS	AC	
RNLI	60.8 (2.2)	74.8 (2.4)	61.2 (2.2)	73.9 (2.6)	0.3 (−4.6 to 5.2)	13.9 (8.1 to 19.8)^b^	12.7 (6.7 to 18.6)^b^	1.3 (−7.1 to 9.6)
SIS-ADL	55.3 (2.3)	66.4 (2.4)	57.0 (2.3)	69.9 (2.6)	−2.6 (−8.1 to 2.8)	11.1 (6.4 to 15.8)^b^	12.9 (8.0 to 17.8)^b^	−1.8 (−8.6 to 5.0)
I-HOPE scores								
Activity	0.73 (0.02)	0.77 (0.02)	0.73 (0.02)	0.78 (0.02)	−0.01 (−0.05 to 0.04)	0.04 (0.003 to 0.08)^c^	0.05 (0.01 to 0.09)^c^	−0.01 (−0.06 to 0.05)
Performance	2.42 (0.10)	3.60 (0.10)	2.65 (0.09)	3.44 (0.11)	−0.04 (−0.24 to 0.16)	1.18 (0.91 to 1.45)^b^	0.79 (0.52 to 1.06)^b^	0.39 (0.01 to 0.77)^c^
Satisfaction	2.24 (0.11)	3.56 (0.12)	2.53 (0.11)	3.33 (0.13)	−0.03 (−0.26 to 0.20)	1.32 (1.01 to 1.63)^b^	0.80 (0.49 to 1.11)^b^	0.52 (0.08 to 0.96)^c^
Barrier severity	67.0 (3.6)	30.5 (3.8)	64.4 (3.5)	38.4 (4.1)	−2.6 (−11.1 to 5.9)	−36.5 (−44.4 to −28.6)^b^	−26.0 (−34.1 to −17.8)^b^	−10.6 (−21.9 to 0.8)

Abbreviations: AC, attentional control; COMPASS, Community Participation Transition After Stroke; I-HOPE, In-Home Occupational Performance Evaluation; RNLI, Reintegration to Normal Living Index; SIS-ADL, Stroke Impact Scale–Activities of Daily Living domain.

^a^ Models were adjusted for race, marital status, living situation, scores on the Geriatric Depression Scale–Short Form, number of days in an inpatient rehabilitation facility, and baseline calendar time.

^b^ *P* < .001.

^c^ *P* < .05.

### Secondary Outcomes

**Figure 3.**
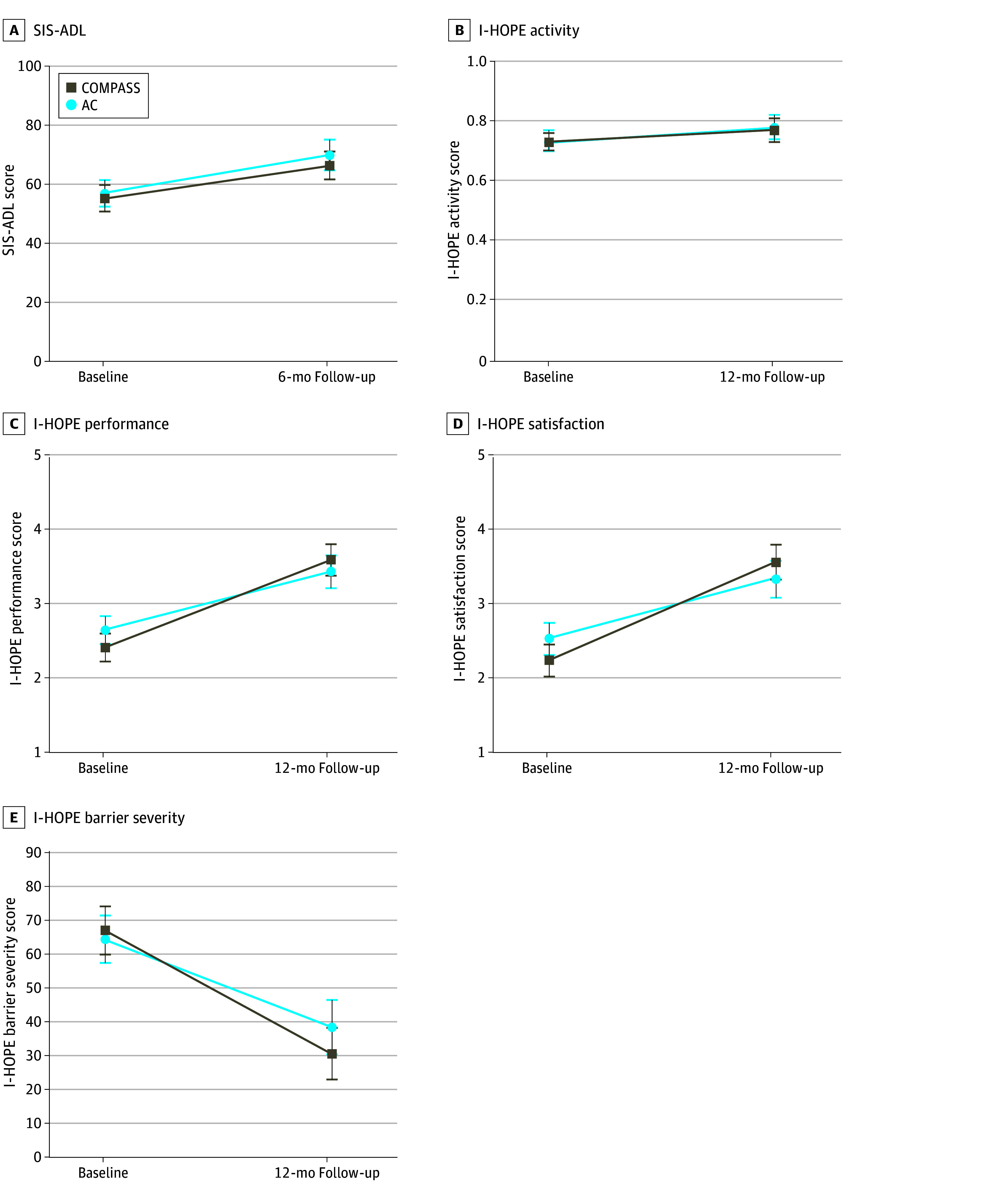
Estimated Marginal Means for Daily Activities and Barrier Severity at Baseline and 12 Months Error bars indicate 95% CIs. AC indicates attentional control; COMPASS, Community Participation Transition After Stroke; I-HOPE, In-Home Occupational Performance Evaluation; SIS-ADL, Stroke Impact Scale–Activities of Daily Living domain.

Mixed-model results are given in Table 2, and estimated marginal means are shown in Figure 3. When using baseline and 12-month data, there were significant improvements in all secondary outcomes over time for both groups. There were significant group × time interactions for the I-HOPE performance and satisfaction scores, indicating greater improvements for COMPASS than AC (between-group differences in changes over time for performance: 0.39; 95% CI, 0.01-0.77, *P* = .046; satisfaction: 0.52; 95% CI, 0.08-0.96, *P* = .02; clinically meaningful differences based on half of the SD were 0.39 and 0.46, respectively). Improvements in I-HOPE barrier severity scores appeared greater among COMPASS participants than AC participants but were not statistically significant (between-group differences in changes over time, −10.6; 95% CI, −21.9 to 0.8; *P* = .07).

When including 4 time points, the interaction of group × time for I-HOPE barrier severity scores was significant (*P* = .003), with the greatest difference from baseline to 6 months (COMPASS mean decrease, −37.5; 95% CI, −43.9 to −31.2; AC mean decrease, −22.3; 95% CI, −28.8 to −15.7; between-group differences in changes, −15.3; 95% CI, −24.4 to −6.2; clinically meaningful difference based on half of the SD was 17). When including all 4 time points in the models, the group × time interaction for SIS-ADL was not statistically significant (*P* = .06), but results suggested greater improvement for COMPASS immediately after the intervention (mean increase, 11.5; 95% CI, 7.6-15.4) than AC (mean increase, 5.8; 95% CI, 1.8-9.8; between-group differences in change over time, 5.8; 95% CI, 0.2-11.3).

All models described above were adjusted for race, marital status, living situation, depression scores, length of stay in an IR facility, and baseline calendar time. We also ran models adding baseline values of outcomes other than the outcome of interest as covariates, and results were similar. However, the group × time interaction in the model for SIS-ADL, including all 4 time points, did reach statistical significance (*P* = .02), with the greatest difference in change between groups immediately after the intervention (COMPASS mean increase, 11.9; 95% CI, 7.9-15.8; AC mean increase, 4.8; 95% CI, 0.8-8.8; between-group difference in change over time, 7.0; 95% CI, 1.4-12.6).

## Discussion

This study compared the efficacy of COMPASS and AC in improving community participation and ADL performance and reducing environmental barriers after stroke. COMPASS and AC participants experienced similar improvements in community participation. The COMPASS participants experienced greater improvements in ADLs than the AC participants immediately after the intervention, but by 12 months, the improvements were similar for both groups. However, the COMPASS participants experienced greater improvements in self-rated performance and satisfaction in ADLs and a greater reduction of environmental barriers during follow-up.

Although improvements in community participation were not greater for COMPASS participants than AC participants, this could have been influenced by the COVID-19 pandemic, when people physically distanced themselves, wore face masks, and stayed home.^72^ The COVID-19 pandemic prevented us from completing community outings with participants. The pandemic reduced in-person social interactions among adults and negatively influenced their activity and well-being,^73,74^ which could have minimized intervention effects.

Daily activity performance, satisfaction, and environmental barrier severity showed greater improvements for COMPASS than AC participants. COMPASS participants showed greater improvement in daily activities (measured by SIS-ADL) immediately after treatment, but both groups demonstrated similar improvements at 12 months. Some spontaneous or natural recovery occurs over time; stroke survivors may benefit from using COMPASS to work on solutions to performing daily activities as soon as they return home instead of waiting 6 to 12 months^21^ to begin adapting to new challenges and reestablishing daily routines after discharge from an IR facility.^20,75^ In general, estimates of changes over time within each group and differences in changes between groups for SIS-ADL (immediately after intervention) and daily activity performance, satisfaction, and environmental barrier scores reached or closely approached clinically meaningful differences (SIS-ADL clinically important difference 5.9^76^; clinically meaningful differences for other outcomes calculated using half of the SD^77^ were 0.39 for performance, 0.46 for satisfaction, and 17 for environmental barriers).^76,77^

### Limitations

This study has several limitations. First, the study occurred during the COVID-19 pandemic, which restricted our ability to examine the effects of COMPASS on community reintegration. Second, we retained only 66.0% of AC participants vs 90.6% of COMPASS participants. Many AC participants dropped out early for numerous reasons and may have needed different retention strategies to remain engaged in the study. Although there was no evidence to suggest that the outcomes differed between dropouts and completers within the AC and COMPASS groups, more AC participants died,^78^ and others may have dropped out due to poorer health than COMPASS participants, which could have weakened findings. Third, we do not know whether AC participants obtained home modifications during this study, which could have affected their participation in daily activities.

## Conclusions

Although the COVID-19 pandemic affected our ability to examine the efficacy of COMPASS on community participation, this randomized clinical trial found that COMPASS reduced environmental barriers and improved self-rated performance of ADLs and satisfaction compared with AC. COMPASS may be a promising strategy to bridge the gap from IR facilities to home by reducing environmental barriers and fostering quicker engagement in ADLs, which may improve stroke survivors’ long-term health outcomes.

## Notes

Supplement 1.
Trial Protocol

Supplement 2.
Data Sharing Statement
