# Supplementary material for: Rehabilitation Transition Program to Improve Community Participation Among Stroke Survivors: A Randomized Clinical Trial
Source: JAMA Netw Open. 2024 Oct 7;7(10):e2437758. doi: 10.1001/jamanetworkopen.2024.37758 (PMC11581659; doi:10.1001/jamanetworkopen.2024.37758)
Supplement: Supplement 1. — Trial Protocol [file jamanetwopen-e2437758-s001.pdf]

COMPASS: A Novel Transition Program to Reduce Disability after Stroke

Study Protocol

Version: 8.3
Original: October 26, 2017
Last Updated: August 7, 2023

**Principal Investigator:**	Susan Stark, PhD, OTR/L<br>Program in Occupational Therapy<br>Washington University School of Medicine<br>St. Louis, Missouri
**Co-Investigators:**	Alexandre Carter, MD<br>Department of Neurology<br>Washington University School of Medicine<br>St. Louis, Missouri
**Biostatistician:**	Yan Yan, MD, PhD<br>Division of Biostatistics<br>Washington University School of Medicine<br>St. Louis, Missouri
**Study Site:**	Washington University School of Medicine<br>4444 Forest Park Avenue<br>St. Louis, MO 63110

Table of Contents

Key Abbreviations ... **5**

Glossary of Terms ... **6**

Version Updates ... **7**

World Health Organization Data Set ... **8**

Organizational Structure and Responsibilities ... **12**

Abstract ... **13**

Methods ... **15**

Research Design Overview ... **15**

Study Participants ... 15

Recruitment ... **16**

Informed Consent ... **16**

Baseline Home Visit (T1) for All Participants ... **17**

Randomization and Blinding ... **18**

Phone Assessment T1b ... **18**

Primary Intervention ... **18**

Defining the Treatment (Table 1) ... 19

Attention Control Group ... 19

Follow-up Period for All Participants ... **20**

Data Collection ... **21**

Outcome Assessments for Baseline & Follow-up Home Visits ... **21**

Demographic Assessments ... **22**

Statistical Analyses ... **23**

Intention-to-Treat Analysis ... **23**

Baseline Analyses ... **23**

Missing Data ... **23**

Primary Analysis ... **23**

Secondary Analyses ... **24**

Safety ... **24**

Process Outcomes ... **24**

Sample Size Calculations ... **24**

Strengths and Limitations ... **25**

Potential Benefits, Risks, and Alternatives ... **25**

Benefits ... **25**

Risks ... **25**

Minimization of Risks and Confidentiality ... **25**

Adverse Event Reporting and Safety Monitoring ... **26**

Premature Study Termination ***27***
Indemnity ***27***
Ethics and Dissemination ***27***

Key Abbreviations

AC	Attention Control
ADLs	Activities of Daily Living
AE	Adverse Event
BI	Barthel Index
BJH	Barnes Jewish Hospital
CGI	Caregiver Interview
COMPASS	Community Participation Transition after Stroke
FIM	Functional Independence Measure
HIPAA	Health Information Portability and Accountability Act
HRPO	Human Research Protection Office
IADLs	Instrumental Activities of Daily Living
ICF	International Classification of Function, Disability and Health
ICH	Intracranial Hemorrhage
I-HOPE	In-Home Occupational Therapy Evaluation
IR	In-patient rehabilitation
IRB	Institutional Review Board
IS	Ischemic Stroke
NIH	National Institutes of Health
NIHSS	National Institute of Health Stroke Scale
OT	Occupational Therapy
PROMIS	Patient Reported Outcomes Measurement Information System
PSS	Perceived Stress Scale
RNLI	Reintegration to Normal Living Index
RCT	Randomized Controlled Trial
SBT	Short Blessed Test
SAS	Statistical Analysis System
SIS	Stroke Impact Scale
SPSS	Statistical Package for the Social Sciences
TRISL	The Rehabilitation Institute of St. Louis

Glossary of Terms

Environmental barriers	Features of the built environment which makes activity performance difficult or unsafe (e.g. lack of hand support by toilet to hold onto while transferring)
Environmental modifications	Changes to the built environment which remove the barrier and compensate for loss in function that a person might experience after a stroke (e.g. installing grab bars on each side of the toilet to give individual hand hold while transferring).
Strategy training	Strategy training is an iterative rehabilitation intervention that promotes goal setting, planning and self-monitoring used to help individuals identity and overcome difficulties on their own.
Tailoring	Tailoring is the process the interventionist uses to adapt the treatment intervention to match and compensate for each participant's pattern of functional loss and unique home and community environments.
Motivational interviewing	Motivational interviewing is a method used to help individuals resolve ambivalent feelings and insecurities to find the internal motivation needed to change behavior. It is a practical empathetic process that takes into consideration the difficulty people face when making life changes, especially after a significant health event like a stroke.
Booster Visit	A visit which occurs months after the initial intervention that is completed to reinforce the strategies learned during the intervention as well as help the participant problem solve any new performance problems which might have arisen after the initial intervention was completed.
Community participation	An individual's involvement his/her own community, as the individual sees fit and meets his/her individual desires and needs.

Version Updates

Date	Version	Description
3/23/18	1.0	Added phone call system, caregiver and therapist participant recruitment and measures, removed Lily Hu and changed Emily Somerville to study coordinator
4/9/2018	2.0	Changed inclusion criteria to include intracranial hemorrhagic stroke as well, per discussion with study neurologist, Dr. Alex Carter.
6/14/2018	3.0	Changed time points caregivers are assessed
10/18/18	4.0	Added transportation option to home visit as staff vehicle. This has been ok'd by Wash U insurance group
11/30/18	5.0	If a person has an extended hospital stay or goes to a SNF for the short term, we will push back their T3 based on the extra days of stay. Normal LOS is considered 3 days.
3/8/19	6.0	Changed our n to 180, based on retention rates
1/2/20	7.0	Decided if T3 is missed by more than 3 months, we will skip T3 and go to T4
3/31/20	7.1	Because of the stay-at-home orders our primary endpoint will be impacted (community participation). In addition, because we can't go into people's homes, we will need to do follow up via phone and won't be able to do the I-HOPE barriers. We will add repeat of the follow-up time points to collect data in the home when we can go back in. Those timepoints will be T2COVID, booster 1&2COVID,T3COVID, T4COVID. At this point, those are 60 days after their original time point (30 days for SAH orders and 30 more days to allow the person to get back into the community after the SAH orders are lifted).
4/1/20	7.2	We will ask our participants if they have access to technology needed for telehealth and are willing to participate in visits remotely using telehealth.
4/9/20	7.3	In order to better understand the impact the COVID-19 pandemic is having on our participants, we will add additional measures to ask all participants, both those who have finished and those are still in follow up. Measures will include: RNLI, SIS, GDS, PROMIS SF Anxiety, NIH Instrumental Support, Pittsburgh Sleep Quality Index, PROMIS SF Social Isolation, 3-Item UCLA Loneliness Scale, and the Connor Davidson Resilience Scale.
6/26/20	7.4	In order to reduce physical contact with participants during the COVID-19 pandemic, we will switch to electronic consenting when at all possible.
7/10/20	7.5	Added that we will continue with e-consenting even after COVID-19 is over
11/11/20	8.0	Added the option for modified assessment process during COVID when participant is not able to leave TRISL for home assessment.
1/6/22	8.1	Added question about participants' COVID-19 vaccination status.
6/22/2023	8.2	Updated contact information for study personnel
8/28/23	8.3	Added statement about waiver of consent to collect chart review data as a follow up metric to identify patient outcomes and for safety monitoring.

World Health Organization Data Set

**Primary Registry and Trial Identifying Number**	ClinicalTrials.gov: NCT03485820
**Date of Registration**	April 2, 2018
**Secondary Identifying Numbers**	IRB ID#: 201705047
**Source(s) of Monetary Support**	Funding for the COMPASS trial is through the National Center for Medical Rehabilitation Research (NCMRR), 1R01HD092398-01
**Primary Sponsor**	Program in Occupational Therapy—Washington University School of Medicine in St. Louis, MO
**Secondary Sponsor(s)**	N/A
**Contact for Public Queries**	Susan Stark, PhD, OTR/L<br>sstark@wustl.edu<br>314-273-4114<br>Program in Occupational Therapy<br>Washington University School of Medicine<br>5232 Oakland Ave<br>St. Louis, MO 63110<br>United States
**Contact for Scientific Queries**	Susan Stark, PhD, OTR/L<br>sstark@wustl.edu<br>314-273-4114<br>Program in Occupational Therapy<br>Washington University School of Medicine<br>5232 Oakland Ave<br>St. Louis, MO 63110<br>United States
**Public Title**	COMPASS: A Novel Transition Program to Reduce Disability after Stroke
**Scientific Title**	COMPASS: A Novel Transition Program to Reduce Disability after Stroke
**Countries of Recruitment**	United States

**Health Condition(s) or Problem(s)**	Stroke in adults
**Intervention(s)**	Study arm 1: Home Modifications<br><br>Study arm 2: Attention Control
**Key Inclusion and Exclusion Criteria**	<u>**Participants with Stroke**</u><br>**Inclusion criteria**:<br>(1) aged ≥50 years<br>(2) acute ischemic stroke (IS) or focal intracranial hemorrhagic (ICH) stroke diagnosis verified by study neurologist Dr. Alexandre Carter<br>(3) independent in activities of daily living (ADLs) prior to stroke (premorbid Modified Rankin Scale Score ≤2)<br>(4) plan to discharge to home<br><br>**Exclusion criteria**:<br>(1) severe terminal systemic disease that limits life expectancy to < 6 months<br>(2) previous disorder (e.g., dementia) that makes interpretation of the self-rated scales difficult or Short Blessed Test (SBT) score of 10 or less (indicating significant cognitive impairment)<br>(3) moderate-to-severe aphasia as determined by the National Institutes of Health Stroke Scale (NIHSS) Best Language rating of 2 or more<br>(4) reside in congregate living facility<br><br><u>**Caregivers**</u><br>**Inclusion criteria**: (1) primary informal (family or friend) caregiver of a stroke patient enrolled in the COMPASS II study, (2) aged ≥18 years<br>**Exclusion criteria**: (1) non-English speaking
**Study Type**	Interventional/Randomized Controlled Trial phase: IIb<br>Allocation: Randomized<br>Intervention model: Parallel assignment<br>Masking: Interventionist not blinded to group, participant not blinded to group, primary outcome assessor blinded to group. PI, rehabilitation staff at The Rehabilitation Institute of St. Louis (TRISL) blinded to participant group assignment.<br><br>Primary purpose: Community reintegration
**Date of First Enrollment**	January 19, 2018
**Target Sample Size**	180

**Recruitment Status**	Closed to Enrollment
**Primary Outcome(s)**	**Specific Aim 1 (Primary)**: We will compare the efficacy of a novel enhanced rehabilitation transition program (COMPASS) and an equivalent dose of attentional control for significant improvements in community participation and ADL performance and a reduction in environmental barriers in the home and community after stroke.<br><br>**Specific Aim 2 (Secondary)**: We will evaluate alternative primary outcome measures of participation, function, patient-reported quality of life, and caregiver burden that permit comparison to other stroke clinical trials and confirm the safety of COMPASS.<br><br>**Specific Aim 3 (Secondary)**: We will evaluate process outcomes such as reach, cost, fidelity, and adherence to aid interpretability of the trial and future implementation.<br><br>**Primary Endpoint**: <u>Reintegration to Normal Living Index (RNLI)</u>. The RNLI measures the extent to which a person is able to resume normal life activities after illness or injury.[1] The 11-item questionnaire quantifies participation (basic self-care, functional mobility, avocational and productive pursuits, travel in the community), with a higher score indicating higher attainment of normal levels of living. The RNLI is a valid and reliable[2] measure that is well-aligned with the primary aim of this trial. The scale demonstrated excellent internal consistency,[3] construct and content validity,[2,3] and interrater reliability.[2] The RNLI is brief and can be administered in person or by telephone.<br><br>**Secondary endpoints**: <u>Stroke Impact Scale (SIS) ADL Domain</u>. The SIS is a stroke-specific assessment of health-related quality of life (participation, ADLs, mobility, hand function, strength, memory, communication, emotion). It has demonstrated high content validity[4] and construct validity.[5] The SIS domains have excellent internal consistency (Cronbach $\alpha = 0.83–0.90$)[6] and test–retest reliability (Interclass correlation range 0.7–0.92),[6] and discriminate across four Rankin levels of stroke severity ($P \leq 0.01$).[6] The SIS ADL domain demonstrated a moderate (0.44) effect size in our pilot.<br><br>The <u>In-Home Occupational Performance Evaluation (I-HOPE)</u> to measure ADL performance and magnitude of environmental barriers in the home. The I-HOPE is a performance-based, multistep, client-centered assessment that evaluates the performance of older adults doing 44 activities in the home. Four subscales measure limitations in daily activities, self-reported performance and satisfaction with performance of problematic activities, and the magnitude of environmental barriers that influence performance. I-HOPE assessment is commonly used in occupational therapy (OT) to provide home modification interventions. The instrument yields four subscales with high internal reliability ($\alpha = 0.77–0.78$) and interrater reliability ($\alpha = 0.94–1.0$)[7] and is sensitive to change in environmental support.<br><br>**Exploratory endpoints**: Our goal is to optimize the design of a phase III multicenter trial; therefore, we will examine additional endpoint measures to evaluate their utility in the trial setting. These measures

include the Caregiver Inventory[8] (CGI; caregiver burden), Perceived Stress Scale,[9] Barthel Index[10] (BI; ADL performance), and Patient-Reported Outcomes Measurement Information System (PROMIS) Physical and Mental Health Scales[11] (health-related quality of life).

**Time Points of Interest**:	Baseline, immediately post-intervention, 6 and 12 months post-stroke

Organizational Structure and Responsibilities

Principal Investigator:
Susan Stark, PhD, OTR/L

Responsibilities include: Managing the operations of the study, ensuring tasks are completed, ensuring compliance with quality assurance requirements (e.g., human participant protection), preparing interim reports and publication of study reports.

Study Coordinators:

Brianna Holden, MS, OTR/L and Emily Somerville, OTD, OTR/L

Responsibilities include: Developing all study materials including the Manual of Procedures and study forms; verifying informed consent from each participant; reporting adverse events (AEs) and serious adverse events (SAEs); recruiting, screening, enrolling, and randomizing participants; following and scheduling participants through study completion; protecting participants' rights; submitting documents to regulatory bodies; developing and implementing quality control procedures, liaison with community partners.

Data Management

Missy Krauss, MPH

Clinical Research Supervisor

Dana Sutter, MSOT

Occupational Therapy Practitioners
Brianna Holden, MS, OTR/L
Meghan Haxton, COTA/L
Becky Bollinger, OTD, OTR/L
Responsibilities include: managing delivery of the intervention in accordance with the study protocol. OTPs will document all visits and data collection.

Data Management Committee:
Susan Stark, PhD, OTR/L
Yan Yan, MD

Responsibilities include: Statistical design of study data verification, developing and implementing data management procedures including the data flow and procedures for data entry, error identification and correction, and preparing quarterly reports on enrollment, participant status (e.g., withdrawals), AEs, and independent safety monitoring body reports.

Data and Safety Monitoring Committee:
Susan Stark, PhD, OTR/L

Responsibilities include: reviewing and evaluating the study data to ensure participant safety, study conduct, progress, and efficacy, and making recommendations regarding the continuation, modification, and termination of the trial.

Abstract

Stroke is a leading cause of disability in the US. Most stroke survivors have difficulty performing daily activities and participating in the community. Efficacy of interventions that address the chronic needs of stroke survivors has been identified as a high priority for stroke research. A gap in care exists at the point of transition from inpatient rehabilitation (IR) to home, when survivors encounter new environmental barriers due to the cognitive and sensorimotor sequelae of stroke. Resolving these barriers and improving independence in the community have potential to significantly improve stroke survivors' long-term morbidity. The proposed study investigates the efficacy and safety of a novel, enhanced rehabilitation transition program to reduce environmental barriers and improve daily activity performance and community participation. Community Participation Transition after Stroke (COMPASS) uses two complementary evidence-based interventions: home modifications and strategy training delivered in the home. Home modifications provide environmental support to compensate for impairments. Strategy training enables patients to identify and prioritize ADL problems, identify barriers to performance, and develop strategies to resolve barriers. The complementary effects of these therapies at a novel point of care offer a robust intervention for a current service gap. The primary aim of this phase IIb, single-blind, parallel-group, randomized controlled trial is to investigate the efficacy of COMPASS during the transition from IR to home. Participants will be randomized to receive either COMPASS or attentional control. We hypothesize that COMPASS participants will demonstrate significant improvements in community participation and ADL performance and a significant reduction in environmental barriers versus control. In preparation for a phase III multicenter trial, we will explore additional candidate study endpoints and establish intervention safety by examining the rates of falls and hospital readmissions. Finally, we will conduct a process evaluation examining outcomes such as reach, cost, fidelity, and adherence to aid interpretability of the trial and implementation. The aims fill critical gaps in stroke rehabilitation evidence by investigating the efficacy, safety, and implementation of an intervention targeting the transition from an acute to a chronic condition. The results of this trial will provide important information about the long-term participation and environmental barriers of stroke survivors. The project has the potential to resolve the significant unmet need of disability after stroke.

Background

Stroke is highly prevalent, costly, and disabling. Stroke is a leading cause of serious long-term disability in the US.[12] Half of stroke survivors are dependent on caregivers to perform their ADLs.[13,14] Unless a solution is identified to improve the long-term outcome of stroke survivors, annual US costs attributed to stroke are projected to increase to $240.67 billion by 2030.[15] Patients report "waiting" at home 6–12 months after discharge for "recovery" before attempting to resume participation in daily activities.[16] The transition from IR to home is an important window of opportunity for intervention.[17,18] Resumption of previous activities immediately after discharge,[19] at a time when people with stroke report struggling to reestablish daily routines,[18] can improve immediate and long-term community reintegration.

Transition home from inpatient rehabilitation (IR) is understudied, but face validity exists for treatments that reduce environmental barriers and improve daily activity performance. A paucity of evidence exists for interventions targeting the transition period from IR to home. In a systematic analysis of the *Guidelines for Adult Stroke Rehabilitation and Recovery,*[20] we mapped COMPASS elements to the evidence recommendations. Transition interventions including environmental modifications (provision of adaptive equipment) and problem-solving approaches like those proposed in COMPASS are rated as having high face validity and low risk but lack rigorous evidence of efficacy. COMPASS also directly addresses goals in the NIH Plan for Rehabilitation Research.[21] The plan has prioritized research that provides an understanding of environmental barriers and individual participation outcomes in real-world settings and specifically calls for research using self-management strategies like strategy training that can be implemented in community settings to achieve patient independence and improve caregiver outcomes.

Reducing environmental barriers improves daily activity performance but is unproven among stroke survivors. Providing environmental support is a potent and immediate strategy to improve daily activity performance. In our systematic review[22] and home modification practice guideline[23] (including 35 articles with levels I–III evidence), strong evidence shows that home modifications improve daily activity performance[24-36] and reduce falls[37-43] among adults and older adults with disabling conditions. Notably, during this review, we did not discover a study of the efficacy of environmental modifications for stroke. That may explain why environmental modification programs are not part of IR, despite face validity that environmental interventions might be an effective approach to reduce excess disability for this population.

Strategy training shows promise in acute IR for stroke but is unproven at the point of transition home. Strategy training enables patients to identify and prioritize daily activity problems and to identify barriers to performance and strategies to resolve the barriers. The approach leads to generalization and learning rather than skill attainment.[44,45] OT scientists have demonstrated the efficacy of guided strategy training interventions for patients with neurological impairments, including acute stroke.[46-52] Strategy training is consistent with new models of inpatient stroke care such as those tested by Skidmore[46-48] but is unproven in transition programs for stroke survivors.

We will provide evidence of the efficacy and safety of a transition program designed to increase the independence of stroke survivors. COMPASS is a manualized intervention that includes pre- and post-discharge visits in the home to remove barriers through home modification and strategy training. The intervention targets community reintegration, or the ability to resume daily activities in the home and community. Our preliminary data provide evidence of acceptability and feasibility for delivery during IR (versus home health, for which regulatory policies and practice patterns preclude a focus on community participation).[53,54] If effective, this program will reduce disability in daily activity performance and improve participation outcomes. We will explore anticipated downstream effects of the intervention including rehospitalization rates and caregiver burden.

The conceptual model of this intervention (Figure 1) is the World Health Organization's International Classification of Functioning, Disability and Health (ICF).[55] The ICF describes the mechanism of COMPASS. Survivors of IS experience decreased cognitive, sensory-motor, and emotional function. After stroke, the home and community environments of survivors pose barriers that prevent successful performance of daily activities and participation. Intervening to remove barriers and enable survivors to use problem-solving strategies to overcome barriers will improve daily activity performance and participation outcomes. This intervention is designed to augment current rehabilitation practice focused at the body structure and function domains. This conceptual model is empirically supported by our recent work exploring the role of environmental barriers in function.[56]

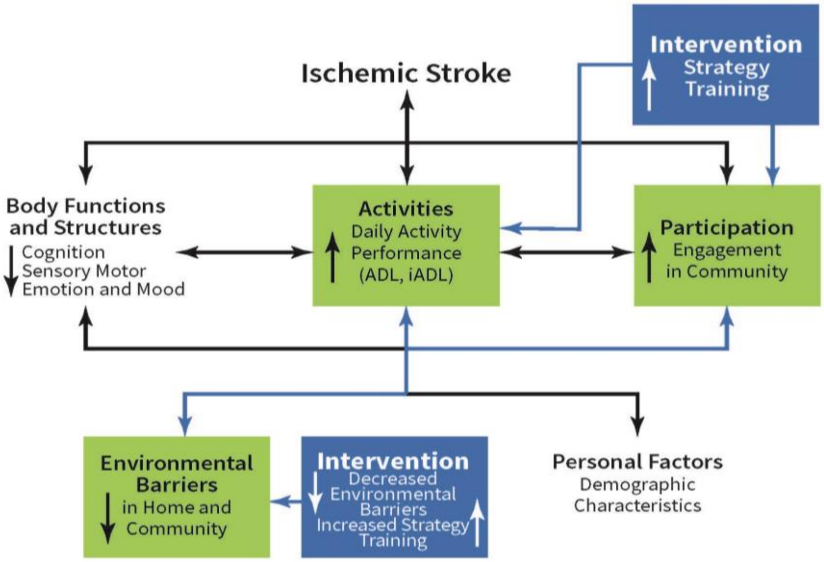

Figure 1. *COMPASS conceptual model adapted from the International Classification of Functioning, Disability and Health*. Blue boxes represent intervention. Blue lines and green boxes represent the hypothesized mechanism of action. By reducing environmental barriers, activity and participation outcomes will improve. We will measure covariates in the domains of body function, personal factors, and health condition.

Methods

Research Design Overview

The objective of this phase IIb RCT is to determine the efficacy and safety of a compensatory intervention designed to improve daily activity performance and participation outcomes in preparation for a definitive trial. We will test the hypothesis that a manualized, theoretically grounded, and empirically supported transition intervention (COMPASS) is superior to an equivalent dose of attentional control (AC) for daily activity performance and participation outcomes with high adherence. The hypothesis is based on our previous work,[35,36] the work of others,[27,57,58] and our preliminary data. We will recruit 180 patients currently undergoing IR for IS or focal ICH and randomize them to receive five in-home sessions (one pre-discharge visit and four post-discharge visits) and two booster contacts of the enhanced rehabilitation transition program or AC. Our primary endpoint of participation and secondary outcome of ADL performance will be assessed by certified blinded raters at baseline, immediately after intervention, and at 6 and 12 months after stroke. Measures of mobility, cognition, and depression, will be used to characterize the clinical phenotype or body structure and function of the participants as important covariates. We will also examine important process outcomes such as cost, satisfaction, adherence, safety, and caregiver burden.

Study Participants

Stroke patient inclusion criteria: (1) aged ≥50 years, (2) acute IS or focal ICH diagnosis, (3) independent ADLs prior to stroke (premorbid Modified Rankin Scale Score ≤2), (4) plan to discharge to home.
Stroke patient exclusion criteria: (1) severe terminal systemic disease that limits life expectancy to < 6 months,

(2) previous disorder (e.g., dementia) that makes interpretation of the self-rated scales difficult or SBT score of 10 or less (indicating significant cognitive impairment), (3) moderate-to-severe aphasia as determined by the NIHSS Best Language rating of 2 or more, (4) reside in congregate living facility.

Caregiver inclusion criteria: (1) primary informal (family or friend) caregiver of a stroke patient enrolled in the COMPASS II study, (2) aged ≥18 years
Caregiver exclusion criteria: (1) non-English speaking

TRISL staff:
Inclusion criteria: (1) occupational therapist, physical therapist, or case manager at TRISL, (2) provided treatment to stroke patient enrolled in the COMPASS II study

Exclusion criteria: (1) Non-English speaking

Recruitment

We will recruit participants through Barnes Jewish Hospital (BJH) and TRISL.

At BJH, potential participants will be screened and referred to the study by the Stroke Patient Access Core. At TRISL, rehabilitation staff and physicians will identify potential participants daily and, with permission, refer to the study team. We will target survivors of IS and focal ICH but not subarachnoid hemorrhage. Outcomes for IS and focal ICH are generally better, but those with subarachnoid hemorrhage are associated with considerably increased mortality and decreased function after stroke.[59]

Informed Consent

Stroke Survivors: A study team member will call all patients/caregivers who meet the inclusion criteria and invite them to participate in the study. The informed consent form will be explained over the phone to all patients interested in participating in the study. For potential participants who have computer access and capability, the formal study consent process will be conducted using a REDCap-based electronic consent form. The consent form has been developed in REDCap, a secure, web-based HIPAA-compliant, data collection platform with a user management system allowing the PI to grant and control varying levels of access to study staff. Potential participants would receive an email with a unique link to review the informed consent form online. After the research team explains the study and answers any question, the potential participants can electronically fill in an "Agree" button, followed by their electronic signature. Upon completion of the consent, participants are presented with the option to download a copy of the executed form. The research team will also e-mail a copy of the executed form to the participant. E-consent versioning will be managed using the e-consent Framework in REDCap. Within the e-consent survey options, we have designated the e-consent version number in this application as e-consent version 1. The PDF's of completed responses will have the timestamp, participant name, and e-consent version number inserted in the footer. Future versions of the e-consent will be created by making a copy of the REDCap form and revising it. The old version would be de-activated upon receiving IRB approval for the new version.

If a participant does not have access to a computer or smart device at the time of consent potential participants will be provided with a copy of the informed consent in person or via email. Once the potential participant has had time to look over the consent form, a study team member will talk with the participant by phone to review the study information and answer any questions. If they choose to continue their participation, they will be asked to sign the consent and return to us either by email (scan or photo). The signed consent form will then be downloaded and retained by research staff. We will instruct participants to keep a copy for themselves.

We will continue to use the electronic consent process even after COVID restrictions have been lifted. All procedures will be the same as described above but with an option for signing in person using the electronic consent on a study provided iPad. This will happen as follows: Once the potential participant has had time to look over the consent form provided to them either via email or paper copy, a study team member will talk with the participant in person to review the study information and answer any questions. If they decide to participate, they will be asked to sign the electronic version of the consent using a study provided iPad. We will

email a copy of the consent form if the participant has a valid email address, otherwise we will download a copy and provide it to the participant.

The following privacy protections will be enacted for all patient communication via email: 1) a test email will be sent to the participant to verify their identify (confirm correct recipient) and that this email will be sent in a secure manner (i.e., [secure] in subject line); 2) The body of the email will instruct the participant to send all information as a response to this thread and to not remove the "[secure]" from the subject line; 3) we will document in our research records that the participant agreed to provide information over email.

In the event participants who consented using the REDCap e-consent would need to be re-consented, we will send the participant a link to the new version to discuss, sign electronically, download, and receive vie email as described above.

During the consent process, participants will: (1) have what the study is about and what is expected of them explained in detail either over the phone or in person when remote is not possible, (2) discuss potential problems that could interfere with participation either over the phone or in person when remote is not possible, (3) have their questions answered either over the phone or in person when remote is not possible, and (4) receive a summary of the study and contact information for the PI and study coordinator. Informed consent to participate in the study will be obtained before any test or measurements are performed. The consent form will be signed by a witness and will be stored in REDCap or paper copies will be stored the office of the PI under double locks. Participants will be advised in the consent form that there is a possibility that their medical research record, including identifying information, may be inspected and photocopied by officials of federal or state government agencies and the Washington University Human Research Protection Office (HRPO).

Caregivers: Primary caregivers of stroke patients will be provided with information on the research study via phone or in person. All procedures mentioned above will be followed for the caregivers as well.

<u>Baseline Home Visit (T1) for All Participants</u>

Prior to randomization, baseline activity assessment will be conducted in the home before discharge by an OT using methods established in our pilot study. The intervention is designed to augment rather than replace usual care, so both groups will receive the in-home assessment (considered best practice). Medically stable participants who are eligible for a therapeutic pass will participate in the home visit. The OT provider will meet the participant and their family at TRISL for car transfer training before departing for the participant's home. The visit will be scheduled to avoid disrupting ongoing inpatient therapy and to accommodate family schedules. Transportation will be provided via study staff vehicle, family vehicle or taxi cab. If the participant is *not* able to transfer safely into a family vehicle or if no family or vehicle is available, we will charter a wheelchair-accessible taxi. If the entrance to the home is not accessible, a temporary ramp will be installed. We were able to install ramps within 48 hours of request in our pilot trial. Onsite, we will use I-HOPE to identify environmental barriers in the home and to establish baseline activity levels. Results of the T1 home assessment (for participants in *each arm*) will be shared with the rehabilitation health care team, because home assessment is considered best practice for patients whose disposition is home.[60]

During COVID, we will have the option to complete a modified initial evaluation if the participant is not allowed to leave TRISL, as indicated by the circumstances (e.g. number of cases in the community, orders issued by the Health Department, or current policies at The Rehabilitation Institute of St. Louis). After the participant consents, the study therapist will use the "Participant Intake Form" to gather information from the TRISL therapists about the current level of functioning of the participant as well as the participant's concerns about going home. The study therapist will then arrange with a family member or friend to meet at the participant's home to complete the modified home visit. During the home visit, the therapist will complete a home safety assessment to evaluate the home environment and will also look specifically at the six therapist priority activities from the I-HOPE (getting in/out house, moving around the house, getting on/off toilet, getting in/out of the shower, getting on/off toilet, and getting in/out of the bed) and identify environmental barriers that will impede the participant's performance of those activities. The therapist will take pictures of the home, including the areas where the six therapist priority activities occur. After the modified home visit is completed, the study

therapist will review the home evaluation with the participant and will share the results of the evaluation with the TRISL therapist team.

If the home evaluation is completed during COVID using the procedures described above, the therapist will meet with the study participants in the treatment group before they discharge from TRISL and review recommendations for home modifications and adaptive equipment. The therapist will facilitate implementing the agreed upon modifications/adaptive equipment prior to discharge from IR following normal study procedures.

Randomization and Blinding

Age is a negative marker for functional recovery after stroke, and functional status is a strong predictor of recovery.[61,62] The Functional Independence Measure (FIM) will be used to determine functional status post-stroke. Randomization will be balanced using the participant's age and FIM score.

Participants will be allocated using a 1:1 ratio via adapted randomization sequences generated *a priori* by the study statistician, Dr. Yan, using a computerized formal probability model. The allocation ratio will be maintained at periodic intervals. Randomization sequence concealment will be achieved by query of the REDCap (Research Electronic Data Capture) system.[63] After baseline assessment (T1) is securely uploaded and locked and stratification variables are entered, the interventionist will elicit the treatment assignment in the field in real time using a secure data connection to REDCap. This will permit prioritization of problems for participants assigned to the treatment group.

Upon randomization, participants will be assigned to an interventionist by the treatment coordinator. Although the results of the home assessments will be shared with the rehabilitation staff, IR staff will be blinded to the group placement so that they do not modify their inpatient or discharge treatment plan. All outcomes will be assessed by a blinded rater.

Phone Assessment T1b

To allow time for participants to adjust and personally assess their participation, T1 participation assessments will be conducted for both groups by telephone 2 days after discharge from IR. A rater who is blinded to allocation will conduct the baseline assessment for the primary, secondary, and exploratory endpoints.

Upon completion of T1b phone assessment, the participant will receive visits 2–6 over an 8-week period. The home modification group will progress from daily activity performance to community participation.

Primary Intervention

COMPASS is a complex intervention that combines two evidence-based treatment strategies at a new point of care (transition from IR; Table 1). The objective of home visits by an OT interventionist is to remediate barriers in the home and community that influence daily activities and community participation. The COMPASS manual fully defines and justifies each element of the intervention and has been iteratively revised during the pilot phase. The treatment will include a set of one pre-discharge[64,65] and four 75-minute post-discharge[66] visits (Table 1). The intervention is followed by two booster sessions. Data from the T1 home barrier assessment, demographic assessments, and rehabilitation staff assessment of functional abilities (e.g., transfers) will be used by the OT interventionist to develop an environmental modification intervention plan. Environmental modifications will be installed before discharge if possible. The intervention (not reimbursable) will be provided at no cost to the participant. Problem areas addressed are participant-specific (tailored), but the process to identify and address the target area is systematic. All participants will receive identical intervention components. The standardized components include assessment, identification of five problematic activities (and environmental barriers), identification of three solutions (for each problem), implementation of a solution set selected by the participant, training, and active practice of daily activities in one's own home and community.

During COVID, if the modified home assessment procedures were used, the I-HOPE will be completed with the participant during the first post-discharge home visit, allowing the participant time to experience life in his/her home after the stroke. Priority activities identified during the I-HOPE will then be addressed during the remaining treatment sessions.

Defining the Treatment (Table 1)

The treatment theory guiding the intervention is a competence-press model that posits that removing environmental barriers (e.g., grab bars near the toilet, using accessible transportation) matched with the patient's pattern of functional loss will improve the outcomes of daily activity performance and participation.[67] The two essential components[68] of COMPASS are (1) home modification and (2) strategy training. Both address barriers in the participant's own home and community environment.[69] The intervention is tailored[70-72] by the interventionist to each participant's pattern of functional loss and unique home and community

Table 1. Experimental Compass Treatment	
Dosage and timing	(1) Assessment session (prior to d/c), four 75-minute visits in the home with OT (over 8 weeks); (2) booster sessions (4 & 5 months)
Model/theory	ICF model; Competence-Press Theory
Two components (evidence-based strategies)	**Home modifications**<br>• Assessment<br>• Participants ID problems in the home<br>• Tailored home modifications; shared decision making to select solutions<br>• Active Practice in context<br>**Strategy training**<br>• Active problem-solving to ID problems participating in the community<br>• Guided discovery to ID barriers and solutions, and implementation<br>• Evaluation of process
Approach	• Dose of home modification begins high and tapers; dose of strategy training begins low and increases<br>• Clinician as partner; caregivers included
Standardized elements of tailored approach	• ID up to 10 in-home problematic activities<br>• ID three solutions (for each in-home problem)<br>• Implement selected solutions in home<br>• In-context training, active practice<br>• ID two problematic community activities<br>• Strategy training to resolve community barriers<br>• Two booster sessions (phone or in person)
d/c=discharge; ICF=International Classification of Functioning Disability, and Health; ID=identify; OT=occupational therapy.	

environments. Tailoring is necessary, given the heterogeneity of environments.[22] The participant will engage in active practice with the OT provider. Home modifications coupled with active practice of daily activities improves functional performance of persons with disabilities.[8,25,27,69,73]

Timing: *Pre-discharge Environmental Modification Intervention.* Using standardized assessment and manualized procedures, environmental modifications will be installed before discharge. The intervention (not reimbursable) will be provided at no cost to the participant. *Post-discharge Activity and Participation Intervention.* On completion of T1b phone assessment, the participant will receive visits 2–5 over an 8-week period. Interventions will progress in complexity from daily activities to community participation based on the participant's goals. A booster session will be provided at 4 and 5 months after intervention over the phone; participants may request a home visit.

Attention Control Group

The control group will experience the same effects of time and attention in the home but no effect on the outcome of interest.[74] Because this study examines the efficacy of a new intervention, there is no opportunity for a usual care arm with dose equivalency. Because 75% of stroke survivors and 78% of caregivers report that their informational needs were not met in the hospital,[75] we developed and piloted a tailored stroke

education program for AC. An OT practitioner will deliver the program in accordance with “Evidence-Based Educational Guidelines for Stroke Survivors after Discharge Home.”[76] Topic order is determined by participants. Four 75-minute sessions will be provided. Topics include stroke symptoms, risk factors and preventing stroke recurrence, nutrition, managing emotions, sleep, fatigue, pain, social support, and sexuality. Written materials from the National Stroke Association and the American Stroke Association are provided. Environmental barriers will not be addressed in the educational sessions.

Follow-up Period for All Participants

Stroke and caregivers: Home visits T2 (immediately after intervention), T3 (6 months after stroke), and T4 (12 months after stroke). The follow-up activity, participation, and process assessments will be conducted in the home. All follow-up assessments will be completed by a blinded rater who did not conduct the T1 evaluation, as new home modifications could reveal group assignment. Distal time points will explore the permanency of any treatment effect on participation. If the participant is not available for in-home assessment, the primary endpoint will be collected by phone. Falls and healthcare utilization are collected ***monthly*** by phone (using an automated system).

For participants who experience a delay in the study timeline (such as hospital admission, travel, short-term admission to a skilled nursing facility, etc.), the timeline will be adjusted accordingly. For example, if someone returns to the hospital during the intervention period and is in the hospital for 4 weeks, their T2 follow-up will be pushed back 4 weeks. If we can’t schedule the T3 for three months past the normal time point for the T3, we will skip the T3 and schedule the T4.

Our primary outcome of the study is community participation. This has been severely limited by the current COVID-19 pandemic stay-at-home orders. We will adjust some of our follow up timepoints for data collection to reflect the SAH orders and allow people a chance to get back into the community before collecting community participation data. Our original timepoints are as follows: T2 (immediate post treatment), T3 (six months post stroke) and T4 (12 months post stroke). We will collect data at those timepoints but we are adding an additional collection time for each of those. Our T2COVID, T3COVID, T4COVID are all 60 days after the original T2/T3/T4 time point to allow for the SAH orders to lift and the participant to resume normal community participation. We will adjust these timepoints if the SAH orders continue longer than anticipated at this point.

Waiver of Consent: Retrospective Chart Review

At this time, we have consent to review medical charts during the enrollment and treatment process. However, we do not have permission to review the record after treatment was completed. Because we have ceased recruiting and all of our participants are finished with their participation in the study intervention and control groups, we no longer have contact with the majority of study participants. We would like to review medical charts (Wash U BJH medical records only) from participants who signed the Wash U COMPASS II consent form. We will collect limited data points, including date of death, hospitalizations, emergency room visits, and SNF/IPR admissions between their date of enrollment in this study and date of final study contact. For the waiver of consent, we will recruit participants already enrolled in this study.

The data collected from this retrospective chart review will be saved in Excel as a limited dataset (with no direct identifiers) on Box in a folder only accessible to the research team and separate from the full REDCap database) to protect the participant’s information from a breach of confidentiality. The results of the research will not affect the clinical care of the individuals because the information is being collected retrospectively after the participant’s completion of the study intervention.

Adherence to the conditions required for an IRB Waiver of Consent are as follows:

1) **The research involves minimal risk,** as the review of the participant’s medical records is for limited information. The likelihood of any adverse effects on the subject’s status, employment, or insurability is exceedingly low. Stringent measures have been implemented to restrict the record review to specific data to minimize the risk of a breach of confidentiality. One research team member trained in EPIC and

HIPAA compliance will access medical records and will only search for and document the above mentioned data. A limited dataset including only an ID number, stroke date, rehab discharge date, date of last visit/last study contact, and dates of death, hospitalization, ER visit, and SNF admission will be saved in an Excel file separate from the REDCap database which contains full study data. No direct identifiers such as names, birth dates, address, phone numbers etc will be saved in this Excel file. Only research team members will have access to look up the ID number in REDCap to view other research data. The Excel file will be stored on a Box folder only accessible by the study team. Data will be retained for six years following project close-out and then will be destroyed. It should be noted that reaching out to subjects for their consent may potentially be seen as an intrusion on their privacy and could lead to undue anxiety or emotional pain amongst participants or their families.

2) **The rights and welfare of the individual would not be adversely affected** because the indicated events have already occurred and been documented in the patient's medical record regardless of the research. The retrospective review of this information takes place after the participant's involvement in the study intervention and therefore does not have any bearing on treatment decisions. Therefore, participants are not denied standard care they are entitled to receive under any circumstances.
3) **The research could not be practicably carried out without a waiver.** Due to the retrospective nature of this data collection, it is not possible to contact all study participants for a complete review of these variables as a follow-up metric. Some participants may be uncontactable due to death, institutionalization, or changes in cognitive status after completing the study intervention.
4) **It would not be appropriate to provide these subjects with information about the results of the research** as the results would have no effect on the participants. The events have already occurred for the participants and they are aware of their occurrence independent of the study. There is no anticipated benefit to the participants that would alter what has already taken place.

Participants who are still enrolled will be re-consented.

Data Collection

<u>Outcome Assessments for Baseline & Follow-up Home Visits</u>

In-Home Occupational Performance Evaluation (I-HOPE). The I-HOPE will be used to measure current activity patterns of participants, identify activities that are difficult but important to them, and identify the environmental barriers that influence those activities (person–environment fit). The I-HOPE is a multi-step assessment that evaluates the performance of older adults doing 42 activities in the home. Using labeled pictures of each home activity (e.g., washing dishes, getting in and out of bed), older adults identify activities difficult for them to perform and self-rate their performance and satisfaction with performing each of the activities. A trained rater (e.g., OT interventionist) observes the older adult performing the activity and quantifies the person–environment fit (or misfit). The instrument yields four subscales: the activity subscale ($\alpha = 0.78$; 6 items); the performance subscale ($\alpha = 0.85$; 38 items); the satisfaction subscale ($\alpha = 0.77$; 8 items); and the environmental barrier subscale ($\alpha = 0.77$; 6 items). Intraclass correlation coefficients were calculated for the I-HOPE subscales on a sample of 10 participants, and scores ranged from 0.94 to 1.0 for raters.[77]

Barthel Index (BI). The BI is a reliable and valid assessment that will be used to assess a person's ability to complete 10 ADLs (feeding, bathing, grooming, dressing, bowel control, bladder control, toileting, chair transfer, ambulation, and stair climbing) and the amount of assistance they need to complete these activities. We will use this information to examine changes in participants' functional performance in this study. There is excellent correlation and agreement for stroke patients between the FIM motor and 10-item Barthel at admission and discharge ($r \geq 0.92$; ICC ≥ 0.83).[78]

Stroke Impact Scale (SIS). The SIS is a health-related quality of life measure that quantifies the impact of stroke on a participant's life, via questionnaire. Fifty-nine items are measured across eight domains: strength, hand function, mobility, ADLs and instrumental activities of daily living (IADLs), emotion, memory, communication, and participation. Performance is self-reported based on the difficulty the participant

experiences with each rater. Each item is scored on a five-point scale, and total domain scores are based on a scale from 0 (poorest outcome) to 100 (best outcome). A final question asks the participant's perception of their recovery post-stroke on a visual analog scale from 0 (no recovery) to 100 (full recovery). Rasch analysis, which is widely used to validate self-report outcome measures, established the SIS's validity and reliability and concluded that it has good psychometric characteristics.[79]

Reintegration to Normal Living Index (RNLI). The RNLI is a disability-related quality of life instrument that will be used to measure participants' satisfaction with their home and community participation and has been validated on a population of community-dwelling individuals with chronic conditions.[2] Participants will read 11 statements related to their monthly activity patterns and assign each one a score based on a 10-point ordinal scale with 1 indicating "does not describe my situation at all" and 10 indicating "fully describes my situation."

Patient-Reported Outcomes Measurement Information System (PROMIS) Physical and Mental Health Scales[11] will be used to measure health-related quality of life. PROMIS is a publicly available system of highly reliable, precise measures of patient-reported health status for physical, mental, and social well-being.

Perceived Stress Scale (PSS).[9] The PSS will be used to measure perceived stress in the caregiver participants. The PSS is a global measure of perceived stress designed for use in community samples. The participant rates feelings and thoughts during the past month using a Likert scale from 0 (never) to 4 (fairly often). The items on the PSS were designed to assess the degree to which respondents find their lives unpredictable, uncontrollable, and overloading.

Caregiver Inventory (CGI)[80]*.* The CGI will be used to measure self-efficacy in caregiving. The CGI is a valid and reliable measure consisting of four subscales—managing medical information (three items), caring for the care recipient (seven items), caring for oneself (five items), and managing difficult interactions and emotions (six items)—and will be collected at all follow-up study visits to examine whether the study had an impact on caregiver burden.

Demographic Assessments

Demographics. Questions such as living situation, education level, and employment status will be used to gather demographic information from the participant. Participants (newly enrolled and those still receiving any in-person study-related visits) will be asked their COVID vaccination status.

Geriatric Depression Scale, Short Form (GDS-SF). The GDS-SF will be used to assess depression levels in stoke participants.[81] The GDS-SF is a 15-item self-report questionnaire specifically designed and validated with the older population and correlates highly with the original 30-item GDS (r=.84).[81] Participants answer yes-or-no questions about their feelings in the past week. Total scores range from 0 to 15, and scores of 5 or more indicate probable depression.

COVID-19 Assessments

In response to the COVID-19 pandemic, we will add additional measures to ask our participants. We will ask current participants these additional measures monthly during the pandemic. If this monthly phone call corresponds with one of their other follow up timepoints, we will ask them at the same time. For participants who have completed the study, we will call them and ask if they are willing to complete the assessments for us as well, so that we can determine if the study intervention has a protective effect during this pandemic and if it allows people to get back to normal life after this pandemic is over. For participants who have completed the study, we will ask them the following measures and also the GDS, SIS and RNLI (described above).

PROMIS Social Isolation. The PROMIS Social Isolation item bank assesses perceptions of being avoided, excluded, detached, disconnected from, or unknown by, others. It is a 4-item short form scored on 1-5 scale; yields a sum score, with lower score indicating less social isolation

3-Item Loneliness Scale. The 3-item loneliness scale is brief, easily administered remotely and the questions have been simplified so people at all education levels can complete it accurately. It is reliable and valid and

take less than a minute to administer. Can be completed remotely. Items are scored on a 3-point likert scale, higher scores indicate more loneliness.

PROMIS Anxiety. PROMIS Anxiety measures anxiety participants are currently experiencing. It is a 4-item scale; measures anxiety participants currently experience on a 1-5 scale; generates a sum score of anxiety with a higher scoring indicating more anxiety.

NIH Toolbox Instrumental Support Scale measures the availability of support for an individual to assist with completing daily tasks; 8-item scale, uses a 5-point likert scale response, scores are summed with higher score indicating more instrumental support.

10-Item Connor-Davidson Resilience Scale will measure resilience or ability to adjust to a stressful situation; consists of 10 items, all answered used a 5-point likert scale; total scores range from 0-40, with higher scores indicating greater resilience.

Healthcare Utilization and Mortality

Retrospective review of the medical records of all randomized participants to collect date of death, hospitalizations, emergency room visits, and skilled nursing facility (SNF)/Inpatient Rehabilitation (IPR) admissions between participants' date of enrollment until date of final study contact as a follow up metric to identify patient outcomes (see waiver of consent).

Statistical Analyses

Intention-to-Treat Analysis

We will perform our analyses using an intention-to-treat paradigm. We will perform exploratory data analysis looking for extreme or otherwise unusual values. Non-normal and heteroscedastic data will be transformed as necessary.

Baseline Analyses

We will use unpaired *t* tests and chi-square tests to compare TØ and baseline characteristics in the two groups for descriptive information (except when statistical assumptions are not met, in which case we may use Wilcoxon or Fisher exact tests).

Missing Data

We expect missing values in outcome measures because of dropout, death, missed assessment, or nonresponse. Our main analysis, a linear mixed-effects model, accommodates missingness due to treatment, prior outcome, or baseline covariates missing at random.[82] Assuming that missing data occur at random, inferences will be valid even if we have differential dropout by intervention arm. If the missing data mechanism is not ignorable (ie, missing is not at random), then mixed-effects selection models or pattern-mixture models will be used.[83]

Primary Analysis

Our primary analysis (testing primary hypothesis) will be based on a linear mixed model using baseline and 12-month RNLIs, accounting for the correlation between a participant's repeated measurements over time. The fixed-effect portion of the model will have the form Yit = β0 + β1 × 12 months + β2Group + β3Group × 12month, in which *Yit* is the RNLI score for participant *i* at baseline (time 0) and 12 months (time 1), and *Group* indicates study arm. In this model, the baseline RNLI is modeled as a dependent variable.[27] For improved precision, the model will be adjusted for baseline covariates including race, sex, depression, and length of hospital stay if an imbalance in covariates between arms is observed in baseline analyses. In this model, β*0* is the mean RNLI score for the control arm at time 0, and β*1* is the change in the mean RNLI from baseline to time 1 for the control

arm; β*2* is the mean RNLI score for the treatment arm at time 0, and β*3* is the change in mean RNLI from time 0 to time 1 for the treatment arm. The primary hypothesis is that the difference in the change in RNLI scores from time 0 to time 1 between arms will be tested by examining β3, which estimates the difference.

Secondary Analyses

For the secondary analyses of the change in SIS score at 1 year and the change in I-HOPE at 1 year, we will use the same approach as for the primary analysis because these two outcomes are also continuous. We have overall type I error control for testing the 1-year change in these three analyses at the design stage. The significance level for testing is 0.016. In addition to comparison of 1-year change, we will extend the model by including scores immediately after intervention and at 6 months to see whether the difference in outcomes is achieved at those time points. Depending on the form of time variable in the model, we will use appropriate regression coefficients or a linear combination of the regression coefficients to determine the difference in change of these scores between arms at certain time points. Interpretation of these results should be cautious because we do not have the type I error controlled for these analyses. Because it is possible that severity of functional impairment after stroke may impact response to treatment, we will analyze impact of functional impairment on response to treatment. We will examine functional impairment by group interaction to examine possible differential intervention effects of functional impairment on community participation and performance of daily activities.

Safety

To determine whether the intervention poses no greater risk than AC, we will examine the differences in numbers of falls and rehospitalizations between groups. The statistical models for count data will be used for analyses of these two outcomes. Using the number of falls as an example, we will fit a Poisson regression model (with overdispersion adjustment if necessary), in which a dummy variable for the intervention arm is used. The regression parameter estimate for this dummy variable is the log of rate ratio of falls for COMPASS versus control arm, and exponentiation of the regression parameter estimate is rate ratio. Using the parameter estimate and its standard error, we can construct a two-sided 95% CI for the rate ratio. We expect the confidence interval for rate ratio will include 1, indicating no significant difference in the fall rate between the two groups.

Process Outcomes

Evaluate acceptability and feasibility to aid in the interpretability of the trial. COMPASS will have high acceptability (80% retention), high fidelity by therapists (95% of elements and 90% of dose delivered), low safety risk (no increased rate of falls or health care use compared with the AC group), and high adherence (80% of modifications in use) at 12 months. We will conduct between-group comparisons of process endpoints collected at each time point (time to first fall, number of injurious falls, health care utilization rate, dosage delivered, and adherence rate) using unpaired *t* tests or chi-square tests. We will compare the characteristics of patients who complete the assigned intervention with those who do not for differences in stroke severity and comorbidities. Descriptive statistics will be used for costs per participant and adherence.

Sample Size Calculations

The study is designed to have 80% of power to reject three null hypotheses of equal mean changes in the primary and secondary endpoints (RNLI, SIS, and I-HOPE) using a two-sided, two-sample, unequal-variance *t* test with overall type I error <0.05. Three alternative mean changes (standard deviation [SD] of change) in intervention and control populations are 15.3 (SD 22.6) versus 1.3 (SD 23.4) for RNLI, 15.7 (SD 16.1) versus 5.6 (SD 9.1) for the SIS ADL domain, and 62.1 (SD 26.1) versus 46.2 (SD 18.8) for I-HOPE. With a 1:1 allocation ratio, 130 patients (65 in each group) are needed for the RNLI outcome, 84 are needed for the SIS outcome, and 100 are needed for the I-HOPE outcome. We will enroll 180 patients to account for a 30% attrition rate. This magnitude of between-group difference is considered clinically meaningful based on prior relevant literature and is achievable based on our pilot study. Sample size calculation includes correlation between baseline and follow-up measures and is based on analysis of change scores, which is equivalent in efficiency to the proposed analytic model. Based on the number of stroke patient participants, we will enroll an equivalent number of caregivers.

Strengths and Limitations

The COMPASS study has important strengths. The intervention is grounded in sound theory and evidence. The intervention has made a significant impact on the ability of people with disability to improve their ability to complete daily activities. The intervention will now be tested as part of a community transition program to help stroke survivors complete daily activities at home, as well as transition back into the community.

The research proposed in this application is innovative because it represents a departure from the status quo by delivering a transition program targeting environmental barriers and providing skill training in the “real world” at the point of discharge from IR. Our preliminary studies suggest that this approach will be highly effective in improving the performance of daily activities and participation outcomes, which could lead to reduced readmission and improved safety.

An additional strength of this study is that it is being conducted in partnership with TRISL, which is managed by HealthSouth, a national health care organization that specializes in providing rehabilitation services for individuals. If this trial is feasible, it can be rolled out nationally with HealthSouth because that partnership already exists and the mechanisms are already in place for a national, multisite trial.

There are a few limitations that exist for this study. The first is low generalizability of findings with only a single site. However, participants at TRISL are comparable nationally regarding demographics, length of stay, and treatment; the only exception is that our sample has a higher percentage of Black patients. In addition, TRISL and HealthSouth have policies in place that limit the amount of contact outside study staff can have with individuals prior to recruitment. In some cases, the policies are even stricter than institutional review board (IRB) policy and make recruitment difficult.

Potential Benefits, Risks, and Alternatives

Benefits

There will be considerable benefits to the participants enrolled in the proposed study. All participants will receive a complete home evaluation provided by a registered and licensed OT interventionist. In addition, participants in the active treatment group will receive free home modifications, while participants in the AC group will have a trained research assistant deliver stroke education.

Risks

Potential risks of research participation—physical, psychological, financial, and legal risks, among others—are considered minimal (45CFR46.404). In-home evaluations and assessments of barriers may result in fatigue or aggravation. In addition, some questions may touch on emotionally sensitive issues that could cause anxiety or other forms of emotional stress. The performance-based testing involves observation of everyday activities, which may result in fatigue or embarrassment. In rare instances, the evaluation protocol could result in a fall. Participants will be told that their involvement in this research study is voluntary and that they may choose not to participate or to withdraw their consent at any time. Withdrawal from the study will not at any time affect the commitment of the clinician to administer care, and there will be no penalty or loss of benefits to which participants are otherwise entitled. Participants who undergo the study visits will be given the option to reschedule the visit or take a break at any time during the study if necessary. There is little legal risk to participating in this research. All research-related information will be kept confidential and accessible only to authorized members of the research team.

Minimization of Risks and Confidentiality

To protect against and minimize potential risks, participants will be carefully screened and evaluated for eligibility by the research coordinator. To avoid or minimize symptoms of fatigue, agitation, or emotional distress due to testing, participants will be instructed to notify the rater or interventionist if they experience any

discomfort. They will also be periodically questioned about their tolerance for the tests/intervention. Testing and interviews will be terminated if participants develop fatigue, agitation, or emotional distress. Participants will be trained in the use of all modifications by a licensed and registered occupational therapist. When photographing the home, only environmental barriers and changes will be collected. Images of participants will not be collected. An ID number will be assigned to each participant. All data collected from a participant will be labeled with the ID number. All participant electronic and hard-copy data will be kept under double-lock protection. All hard-copy forms that contain personal identifiers (e.g., name, address, phone number) will be stored in a separate, locked file drawer under double-lock protection. No publication or presentation of the study data will uniquely identify or provide sufficient information to uniquely identify participants.

Risks during the home visit will be minimized by having licensed and trained interventionists available to monitor safety during the home assessment. If needed, an additional trained assistant will accompany the interventionist and family on the home visit. Gait belts will be used at all times while in the home. If needed, temporary ramps will be installed to facilitate entry

To guard against unauthorized data access, all shared-use computer systems at the Washington University School of Medicine are protected with passwords, which are changed at 4-month intervals. Only individuals with a particular "need to know" status are given access, and system privileges are carefully restricted. All personal computers to be used in the Administrative Unit are located within a secure area, and the system is locked when not in use. SAS and SPSS software packages will be used for data management and analysis. Datasets generated from these programs will be password protected, which will make accessing study data difficult even in the event that unauthorized computer access occurs. Systems connected to the Ethernet are carefully controlled, and all systems without Ethernet access control are insulated from the backbone by bridges or routers. The Ethernet cable itself is routed only through secure passageways.

Data are directly entered into a REDCap database. REDCap servers are securely housed in an onsite, limited-access data center managed by the Division of Biostatistics at Washington University. All Web-based information transmission is encrypted. All data are stored on a private, firewall-protected network. All users are given individual user IDs and passwords, and their access is restricted on a role-specific basis. REDCap was developed specifically around HIPAA security guidelines and is implemented and maintained according to Washington University guidelines. Study data will be collected via tablet in the field and managed using REDCap electronic data-capture tools hosted at Washington University. REDCap is a secure, Web-based application designed to support data capture for research studies.

In order to complete the automated phone calls, first names and phone numbers will be shared with Twilio, an online communication software designed to make automated phone call surveys. To complete the call, Twilio will access first names and phone numbers via REDCap. Survey responses will be temporarily stored by Twilio, transferred into Washington University's secure databases (REDCap), and deleted from Twlilio as soon as possible.

Adverse Event Reporting and Safety Monitoring

All SAEs will be reported to the HRPO in the following time frames: (a) death–immediately, (b) life-threatening–within 7 calendar days, (c) all other SAEs–within 15 calendar days using the Electronic Serious Adverse Event Reporting System. Should an SAE occur that increases the risk to the participants, the study will be stopped, an investigation will be conducted, and a findings report will be generated before the study is resumed.

Dr. Stark will be responsible for reviewing study progress and outcomes including recruitment, data quality, safety, and efficacy. Quarterly reports will be reviewed by the study investigators. Because risk in the proposed study is considered minimal, the data monitoring plan will include continuous, close monitoring by the study investigator with prompt reporting of any AEs. Given the small number of subjects undergoing treatment, problems will become more readily apparent through close monitoring of individual participants. In this study, Dr. Stark will monitor the study for AEs, adherence to the protocol, and safety.

Premature Study Termination

Preliminary study data will be monitored by the data management team for any potentially harmful outcomes. If interim data raise significant safety concerns, the trial will be ended early.

Indemnity

Washington University School of Medicine is responsible for any non-negligent damage incurred as a result of participating in the COMPASS Trial. The indemnity is renewed on an annual basis. Washington University School of Medicine assures that it will continue renewal of the indemnity for the duration of the trial.

Ethics and Dissemination

This protocol and the template informed consent forms will be reviewed and approved by the Washington University IRB with respect to scientific content and compliance with applicable research and human subjects regulations. All study personnel involved in the conduct of this research will receive the required education on the protection of human participant rights.

On publication of the study results, participants will be invited to attend a community meeting, during which the results of the study will be reported. The information will be repeated during three community sessions to be held during daylight hours. Participants will receive a mailing announcing the meetings and summarizing the study findings. The location of the meetings will be in a fully accessible auditorium with accessible parking and access to public transportation. A written report will be distributed, and the results will be presented by the study investigators, followed by a question-and-answer period. Refreshments will be served, and participants and their family members will be thanked for their generous support of the project.

References

1. Wood-Dauphinee S, Williams JI. Reintegration to normal living as a proxy to quality of life. Journal of Chronic Diseases 1987;40:491-9.
2. Wood-Dauphinee S, Opzoomer M, Williams J, Marchand B, Spitzer W. Assessment of global function: The Reintegration to Normal Living Index. Archives of Physical Medicine and Rehabilitation 1988;69:583-90.
3. Bluvol A, Ford-Gilboe M. Hope, health work and quality of life in families of stroke survivors. Journal of Advanced Nursing 2004;48:322-32.
4. Duncan PW, Wallace D, Studenski S, Lai SM, Johnson D. Conceptualization of a new stroke-specific outcome measure: The Stroke Impact Scale. Topics in Stroke Rehabilitation 2001;8:19-33.
5. Duncan PW, Bode RK, Lai SM, Perera S. Rasch analysis of a new stroke-specific outcome scale: The Stroke Impact Scale. Archives of Physical Medicine and Rehabilitation 2003;84:950-63.
6. Duncan PW, Wallace D, Lai SM, Johnson D, Embretson S, Laster LJ. The stroke impact scale version 2.0 evaluation of reliability, validity, and sensitivity to change. Stroke 1999;30:2131-40.
7. Stark SL, Somerville EK, Morris JC. In-Home Occupational Performance Evaluation (I-HOPE). The American Journal of Occupational Therapy 2010;64:580-9.
8. Richards LG, Latham NK, Jette DU, Rosenberg L, Smout RJ, DeJong G. Characterizing occupational therapy practice in stroke rehabilitation. Archives of Physical Medicine and Rehabilitation 2005;86:51-60.
9. Cohen S, Kamarck T, Mermelstein R. A global measure of perceived stress. Journal of Health and Social Behavior 1983;24:386-96.
10. Hsieh C-L, Hsueh I-P, Chiang F-M, Lin P-H. Inter-rater reliability and validity of the action research arm test in stroke patients. Age and Ageing 1998;27:107-13.
11. Hays RD, Bjorner JB, Revicki DA, Spritzer KL, Cella D. Development of physical and mental health summary scores from the patient-reported outcomes measurement information system (PROMIS) global items. Quality of Life Research 2009;18:873-80.
12. Roger VL, Go AS, Lloyd-Jones DM, et al. Heart disease and stroke statistics-2011 Update: A report from the American Heart Association. Circulation 2011;123:e18-e209.
13. Patel AT, Duncan PW, Lai S-M, Studenski S. The relation between impairments and functional outcomes poststroke. Archives of Physical Medicine and Rehabilitation 2000;81:1357-63.
14. Jorgensen HS, Nakayama H, Raaschou HO, Vive-Larsen J, Stoier M, Olsen TS. Outcome and time course of recovery in stroke. Part II: Time course of recovery. The copenhagen stroke study. Archives of physical medicine and rehabilitation 1995;76:406-12.
15. Ovbiagele B, Goldstein LB, Higashida RT, et al. Forecasting the future of stroke in the United States a policy statement from the American Heart Association and American Stroke Association. Stroke 2013;44:2361-75.
16. Kirkevold M. The unfolding illness trajectory of stroke. Disability and Rehabilitation 2002;24:887-98.
17. Michels N. The transition from hospital to home: an exploratory study. Home Health Care Services Quarterly 1988;9:29-44.
18. Rittman M, Faircloth C, Boylstein C, et al. The experience of time in the transition from hospital to home following stroke. Journal of Rehabilitation Research and Development 2004;41:259-68.
19. Rochette A, Bravo G, Desrosiers J, St-Cyr/Tribble D, Bourget A. Adaptation process, participation and depression over six months in first-stroke individuals and spouses. Clinical Rehabilitation 2007;21:554-62.
20. Winstein CJ, Stein J, Arena R, et al. Guidelines for adult stroke rehabilitation and recovery: A guideline for healthcare professionals from the American Heart Association/American Stroke Association. Stroke 2016;47:e98-e169.
21. Eunice Kennedy Shriver National Institute of Child Health and Human Development and the NIH Medical Rehabilitation Coordinating Committee. National Institutes of Health Research Plan on Rehabilitaiton. Bethesda, MD: National Institutes of Health; 2016.

22. Stark S, Keglovits, M., Lieberman, D., and Arbesman, M. Effectiveness of home modification interventions on participation for community-dwelling adults and older adults: A systematic review. American Journal of Occupational Therapy (in press).
23. Siebert C, Smallfield S, Stark S. Occupational Therapy Practice Guidelines for Home Modifications: AOTA Press; 2014.
24. Fange A, Iwarsson S. Changes in ADL dependence and aspects of usability following housing adaptation--a longitudinal perspective. Am J Occup Ther 2005;59:296-304.
25. Gitlin LN, Corcoran M, Winter L, Boyce A, Hauck WW. A randomized, controlled trial of a home environmental intervention: Effect on efficacy and upset in caregivers and on daily function of persons with dementia. Gerontologist 2001;41:4-14.
26. Gitlin LN, Miller KS, Boyce A. Bathroom modifications for frail elderly renters: Outcomes of a community-based program. Technology and Disability 1999;10:141-9.
27. Gitlin LN, Winter L, Dennis MP, Corcoran M, Schinfeld S, Hauck WW. A randomized trial of a multicomponent home intervention to reduce functional difficulties in older adults. J Am Geriatr Soc 2006;54:809-16.
28. Graff MJ, Vernooij-Dassen MJ, Thijssen M, Dekker J, Hoefnagels WH, Rikkert MG. Community based occupational therapy for patients with dementia and their care givers: Randomised controlled trial. BMJ (Clinical research ed) 2006;333.
29. Hagsten B, Svensson O, Gardulf A. Early individualized postoperative occupational therapy training in 100 patients improves ADL after hip fracture: a randomized trial. Acta Orthop Scand 2004;75:177-83.
30. Mann WC, Ottenbacher KJ, Fraas L, Tomita M, Granger CV. Effectiveness of assistive technology and environmental interventions in maintaining independence and reducing home care costs for the frail elderly. A randomized controlled trial. Archives of family medicine 1999;8.
31. Petersson I, Kottorp A, Bergstrom J, Lilja M. Longitudinal changes in everyday life after home modifications for people aging with disabilities. Scand J Occup Ther 2009;16:78-87.
32. Petersson I, Lilja M, Hammel J, Kottorp A. Impact of home modification services on ability in everyday life for people ageing with disabilities. J Rehabil Med 2008;40:253-60.
33. Pighills AC, Torgerson DJ, Sheldon TA, Drummond AE, Bland JM. Environmental assessment and modification to prevent falls in older people. J Am Geriatr Soc 2011;59:26-33.
34. Tomita MR, Mann WC, Stanton K, Tomita AD, Sundar V. Use of Currently Available Smart Home Technology by Frail Elders: Process and Outcomes. Topics in geriatric rehabilitation 2007;23:24-34.
35. Stark SL. Removing environmental barriers in the homes of older adults with disabilities improves occupational performance. Occupation Participation and Health 2004;24:32-9.
36. Stark SL, Landsbaum A, Palmer JL, Somerville EK, Morris JC. Client-centered home modifications improve daily activity performance of older adults. Canadian Journal of Occupational Therapy 2009;76 Spec No:235-45.
37. Campbell AJ, Robertson MC, La Grow SJ, et al. Randomised controlled trial of prevention of falls in people aged > or =75 with severe visual impairment: the VIP trial. BMJ 2005;331:817.
38. Clemson L, Cumming RG, Kendig H, Swann M, Heard R, Taylor K. The effectiveness of a community-based program for reducing the incidence of falls in the elderly: A randomized trial. J Am Geriatr Soc 2004;52:1487-94.
39. Close J, Ellis M, Hooper R, Glucksman E, Jackson S, Swift C. Prevention of falls in the elderly trial (PROFET): a randomised controlled trial. Lancet 1999;353:93-7.
40. Cumming RG, Thomas M, Szonyi G, et al. Home visits by an occupational therapist for assessment and modification of environmental hazards: a randomized trial of falls prevention. J Am Geriatr Soc 1999;47:1397-402.
41. Davison J, Bond J, Dawson P, Steen IN, Kenny RA. Patients with recurrent falls attending Accident & Emergency benefit from multifactorial intervention-a randomised controlled trial. Age and Ageing 2005;34:162-8.

42. Huang T-T, Acton GJ. Effectiveness of Home Visit Falls Prevention Strategy for Taiwanese Community-Dwelling Elders: Randomized Trial. Public Health Nursing 2004;21:247-56.
43. Pardessus V, Puisieux F, Di Pompeo C, Gaudefroy C, Thevenon A, Dewailly P. Benefits of home visits for falls and autonomy in the elderly: a randomized trial study. American journal of physical medicine & rehabilitation / Association of Academic Physiatrists 2002;81:247-52.
44. Meichenbaum D. Cognitive behaviour modification. Cognitive Behaviour Therapy 1977;6:185-92.
45. Fitts PM, Posner MI. Human Performance. Brooks. Cole, Belmont, CA 1967.
46. Skidmore ER, Holm MB, Whyte EM, Dew MA, Dawson D, Becker JT. The feasibility of meta-cognitive strategy training in acute inpatient stroke rehabilitation: case report. Neuropsychological rehabilitation 2011;21:208-23.
47. Skidmore ER, Dawson DR, Whyte EM, et al. Developing complex interventions: lessons learned from a pilot study examining strategy training in acute stroke rehabilitation. Clinical rehabilitation 2014;28:378-87.
48. Skidmore ER, Whyte EM, Butters MA, Terhorst L, Reynolds CF. Strategy training during inpatient rehabilitation may prevent apathy symptoms after acute stroke. PM&R 2015;7:562-70.
49. Wolf TJ, Doherty M, Kallogjeri D, et al. The feasibility of using metacognitive strategy training to improve cognitive performance and neural connectivity in women with chemotherapy-induced cognitive impairment. Oncology 2016;91:143-52.
50. Dawson DR, Gaya A, Hunt A, Levine B, Lemsky C, Polatajko HJ. Using the cognitive orientation to occupational performance (CO-OP) with adults with executive dysfunction following traumatic brain injury. Canadian Journal of Occupational Therapy 2009;76:115-27.
51. Goverover Y, Johnston MV, Toglia J, DeLuca J. Treatment to improve self-awareness in persons with acquired brain injury. Brain Injury 2007;21:913-23.
52. Toglia JP. Generalization of treatment: A multicontext approach to cognitive perceptual impairment in adults with brain injury. American Journal of Occupational Therapy 1991;45:505-16.
53. Toto PE. Success through teamwork in the home health setting: The role of occupational therapy. Home Health Care Management and Practice 2006;19:31-7.
54. Boutin-Lester P, Gibson RW. Patients perceptions of home health occupational therapy. Australian Occupational Therapy Journal 2002;49:146-54.
55. WHO. International Classification of Functioning, Disability and Health (ICF). Geneva: World Health Organization; 2001.
56. Hammel J, Magasi S, Heinemann A, et al. Environmental barriers and supports to everyday participation: a qualitative insider perspective from people with disabilities. Archives of Physical Medicine and Rehabilitation 2015;96:578-88.
57. Nikolaus T, Bach M. Preventing falls in community-dwelling frail older people using a home intervention team (HIT): results from the randomized Falls: HIT trial. Journal of the American Geriatrics Society 2003;51:300-5.
58. Wilson DJ, Mitchell JM, Kemp BJ, Adkins RH, Mann W. Effects of assistive technology on functional decline in people aging with a disability. Assist Technol 2009;21:208-17.
59. Andersen KK, Olsen TS, Dehlendorff C, Kammersgaard LP. Hemorrhagic and ischemic strokes compared stroke severity, mortality, and risk factors. Stroke 2009;40:2068-72.
60. Duncan PW, Zorowitz R, Bates B, et al. Management of adult stroke rehabilitation care: A clinical practice guideline. Stroke 2005;36:e100-e43.
61. Jongbloed L. Prediction of function after stroke: A critical review. Stroke 1986;17:765-76.
62. Weimar C, Konig IR, Kraywinkel K, Ziegler A, Diener HC. Age and National Institutes of Health Stroke Scale Score within 6 hours after onset are accurate predictors of outcome after cerebral ischemia: Development and external validation of prognostic models. Stroke 2004;35:158-62.
63. Harris PA, Taylor R, Thielke R, Payne J, Gonzalez N, Conde JG. Research electronic data capture (REDCap)-A metadata-driven methodology and workflow process for providing translational research informatics support. Journal of Biomedical Informatics 2009;42:377.

64. Mountain G, Pighills A. Pre-discharge home visits with older people: Time to review practice. Health & Social Care in the Community 2003;11:146-54.
65. Barras S. A systematic and critical review of the literature: The effectiveness of occupational therapy home assessment on a range of outcome measures. Australian Occupational Therapy Journal 2005;52:326-36.
66. Björkdahl A, Nilsson AL, Grimby G, Sunnerhagen KS. Does a short period of rehabilitation in the home setting facilitate functioning after stroke? A randomized controlled trial. Clinical Rehabilitation 2006;20:1038-49.
67. Lawton M, Namehow L. Ecology and the aging process. In: Eisdorfer CL, Lawton MP, eds. Psychology of Adult Development and Aging. Washington DC: American Psychological Association; 1973:619-74.
68. Whyte J, Hart T. It's more than a black box; It's a Russian doll: Defining rehabilitation treatments. American Journal of Physical Medicine & Rehabilitation 2003;82:639-52.
69. Dobkin BH. Strategies for stroke rehabilitation. The Lancet Neurology 2004;3:528-36.
70. Wagner SL, Davis EHC, Gouthous L, Wallace J, LoGerfo M, Kent D. Preventing disability and managing chronic illness in frail older adults: A randomized trial of a community-based partnership with primary care. J Am Ger Soc 1998;46:1191-8.
71. Schulz R, Burgio L, Burns R, et al. Resources for enhancing Alzheimer's caregiver health (REACH): Overview, site-specific outcomes, and future directions. The Gerontologist 2003;43:514-20.
72. Kreuter MW, Skinner CS. Tailoring: What's in a name? Health Educ Res 2000;15:1-4.
73. Neville-Jan A, Piersol C, Kielhofner G, Davis K. Adaptive equipment. Occupational Therapy In Health Care 1992;8:3-18.
74. Sutherland ER. Sham procedure versus usual care as the control in clinical trials of devices: Which is better? Proc Am Thorac Soc 2007;4:574-6.
75. Eames S, McKenna K, Worrall L, Read S. The suitability of written education materials for stroke survivors and their carers. Topics in Stroke Rehabilitation 2003;10:70-83.
76. Ostwald SK, Davis S, Hersch G, Kelley C, Godwin KM. Evidence-based educational guidelines for stroke survivors after discharge home. The Journal of Neuroscience Nursing: Journal of the American Association of Neuroscience Nurses 2008;40:173.
77. Stark S, Somerville E, Morris J. In-Home Occupational Performance Evaluation. American Journal of Occupational Therapy in press.
78. Hsueh I-P, Lee M-M, Hsieh C-L. Psychometric characteristics of the Barthel activities of daily living index in stroke patients. Journal-formosan medical association 2001;100:526-32.
79. Lin K-C, Fu T, Wu C-Y, Hsieh Y-W, Chen C-L, Lee P-C. Psychometric comparisons of the stroke impact scale 3.0 and stroke-specific quality of life scale. Quality of Life Research 2010;19:435-43.
80. Merluzzi TV, Philip EJ, Vachon DO, Heitzmann CA. Assessment of self-efficacy for caregiving: The critical role of self-care in caregiver stress and burden. Palliative and Supportive Care 2011;9:15-24.
81. Yesavage JA, Sheikh JI. Geriatric Depression Scale (GDS) Recent Evidence and Development of a Shorter Version. Clinical gerontologist 1986;5:165-73.
82. Fitzgmaurice G, Laird N, Ware J. Applied longitudinal analysis. Hoboken. NJ: Wiley; 2004.
83. Little R, D'Agostino R, Dickersin K, et al. The Prevention and Treatment of Missing Data in Clinical Trials. Pannel on Handling Missing Data in Clinical Trials: The National Academies Press; 2010.
