# Supplementary material for: Rehabilitation Transition Program to Improve Community Participation Among Stroke Survivors: A Randomized Clinical Trial
Source: JAMA Netw Open. 2024 Oct 7;7(10):e2437758. doi: 10.1001/jamanetworkopen.2024.37758 (PMC11581659; doi:10.1001/jamanetworkopen.2024.37758)
Supplement: Supplement 2. — Data Sharing Statement [file jamanetwopen-e2437758-s002.pdf]

Data Sharing Statement

Bollinger. Rehabilitation Transition Program to Improve Community Participation Among Stroke Survivors. *JAMA Netw Open*. Published October 07, 2024.
doi:10.1001/jamanetworkopen.2024.37758

Data
Data available: Yes
Data types: Deidentified participant data, Data dictionary
How to access data: Data are available from the corresponding author (sstark@wustl.edu) upon request.
When available: With publication

Supporting Documents
Document types: None

Additional Information
Who can access the data: Researchers whose proposed use of the data has been approved.
Types of analyses: For any purpose.
Mechanisms of data availability: With a signed data access agreement.
Any additional restrictions: N/A
